# Association between blooming time and climatic adaptation in *Prunus mume*

**DOI:** 10.1002/ece3.5894

**Published:** 2019-12-20

**Authors:** Ting Shi, Wenjie Luo, Hantao Li, Xiao Huang, Zhaojun Ni, Haidong Gao, Shahid Iqbal, Zhihong Gao

**Affiliations:** ^1^ Nanjing Agricultural University Nanjing China; ^2^ Guangdong Provincial Key Laboratory for Plant Epigenetics College of Life Sciences and Oceanography Shenzhen University Shenzhen China; ^3^ Genepioneer Biotechnologies Co. Ltd Nanjing China

**Keywords:** blooming time, climatic adaptation, genome resequencing, population genetics, *Prunus mume*

## Abstract

*Prunus mume* Sieb. et Zucc. is an important fruit crop of the subtropical region, originating in China. It blooms earlier than other deciduous fruit trees, but different regions have different blooming periods. The time of anthesis is related to the dormancy period, and a certain amount of chilling promotes bud break and blooming. To identify the relationship between blooming time and the climatic adaptation of *P. mume* cultivars in China, the nuclear and chloroplast genomes of 19 cultivars from the main cultivation areas of *P. mume* in China were resequenced. The average depth of coverage was 34X–76X, and a total of 388,134 single nucleotide polymorphisms were located within the coding regions of the gene (CDs). Additionally, the 19 cultivar accessions were divided into three groups based on their blooming time: early, mid, and late. Associated with the blooming time groups, 21 selective sweep regions were identified, which could provide evidence supporting the possible model of *P. mume* domestication originating due to natural selection. Furthermore, we identified a flowering gene, *FRIGIDA‐LIKE 3* (*FRL3*), seems to affect the blooming time and the climatic adaptation of *P. mume* cultivars. This study is a major step toward understanding the climatic adaptation of *P. mume* cultivars in China.

## Graphical abstract

Twenty‐one selective sweep regions were identified by genome resequencing from 19 *Prunus mume* accessions, which could provide evidence supporting the model of *P. mume* domestication originating due to natural selection. Furthermore, we identified a flowering gene, *FRIGIDA‐LIKE 3* (*FRL3*), seems to affect the chilling requirements and the climate adaptation of *P. mume* cultivars. This study is a major step toward understanding the climate adaptation of *P. mume* cultivars in China.

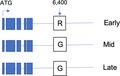

## 1 INTRODUCTION

*Prunus mume* Sieb. et Zucc. is a member of the Rosaceae family, a culturally important deciduous fruit tree in east Asia, including Japan, Korea, and southern China (Chu, 1999). It is mainly used for making liqueurs, pickles, and sauces (Adachi et al., 2007). *P. mume* originates in China and has been domesticated for thousands of years (Chu, 1999; Li, Chen, & Zhang, 2007). The area of Hengduan Mountain is considered the center of natural distribution and genetic diversity of *P. mume* (Chu, 1999). Moreover, the wild species were found in south China such as Guangdong and Fujian (Chu, 1999; Li, 2010).

*Prunus mume* blooms earliest in the spring, with a shorter dormancy period among all deciduous fruit trees. The seasonal dormancy is crucial for winter survival and regulation of timely growth activity of perennial plants in temperate and cold climates (Arora, Rowland, & Tanino, 2003). Bud dormancy has been classified into three categories: paradormancy, endodormancy, and ecodormancy (Cooke, Eriksson, & Junttila, 2012; Faust, Liu, Millard, & Stutte, 1991). Chilling requirements affects bud break and flowering because of its stimulation toward dormancy. *P. mume* has a wide range of chilling requirements from 479 CU to 1,323 CU corresponding with cultivars from Southern China to Northern China (Gao et al., 2012). The chilling requirements of the varieties in southern China such as Guangdong and Fujian province are early while that of varieties in central China such as Jiangsu and Zhejiang province are late. The varied blooming time of varieties may be due to different climatic conditions in different regions (Zhuang, Shi, Gao, Zhang, & Zhang, 2013).

*DORMANCY‐ASSOCIATED MADS box6* (*PmDAM6*), a MADS box gene, was a candidate for growth inhibition and dormancy‐controlling gene (Sasaki et al., 2011). *PmDAM3*, *PmDAM5*, and *PmDAM6* expressions are closely associated with dormancy release in both flower and vegetative buds. PmDAM1 and PmDAM6 could form heteromeric complexes with C‐repeat binding factor 5 (CBF5), a cold response signal factor. PmCBF1, PmCBF3, and PmDAM4 recognized the promoter of *PmDAM6* by the alternative binding sites. Moreover, PmDAM6 could interact with a homolog of SUPPRESSOR OF OVEREXPRESSION OF CONSTANS1 (PmSOC1; Kitamura, Takeuchi, Yamane, & Tao, 2016). The interactions of these genes were key factors for early dormancy release and blooming time in *P. mume*, which made it sensitive to temperature changes, resulting in short dormancy and early flowering (Kitamura et al., 2016; Zhao et al., 2018). Other than these regulators, there are many other genes have been reported to be punitively involved in dormancy release, such as RGL2 (Lv, Huo, Wen, Gao, & Khalil‐Ur‐Rehman, 2018) and hormone‐related genes (Wen et al., 2016). PmRGL2 (REPRESSOR‐OF‐GA‐LIKE2), a DELLA protein, could play a negative role in bud dormancy release by regulating the GA biosynthetic enzymes (Lv et al., 2018). Gibberellin (GA 4) has a significant effect on promoting dormancy release in flower buds of *P. mume* (Zhuang, Gao, et al., 2013; Zhuang et al., 2015). Abscisic acid (ABA) is another plant hormone involved in regulating the onset of dormancy and in maintaining the dormant state (Karssen, Brinkhorst‐van der Swan, Breekland, & Koornneef, 1983; Wen et al., 2016). Furthermore, the QTL analyses localized the significant QTLs controlling leaf bud chilling requirements and heat requirements, leafing date, and PmDAM6 expression in leaf buds to a region in linkage group LG4, which suggests that this locus controls dormancy release, bud break, and PmDAM6's downregulation in *P. mume* leaf buds (Kitamura et al., 2018). QTLs for chilling requirements and blooming time were identified on the LG4 of almond (Sánchez‐Pérez, Dicenta, & Martínez‐Gómez, 2012) and sweet cherry (Castède et al., 2014). The QTLs for apricot blooming initiation time were located on LG1 and LG4 (Dirlewanger et al., 2012; Olukolu et al., 2009; Socquet‐Juglard et al., 2013). In peach, major QTLs for chilling requirements and blooming time were detected on LG1, LG4, and LG7 (Bielenberg et al., 2015; Fan et al., 2010; Zhebentyayeva et al., 2014). Genome‐wide association studies for CR in peach identified seven association peaks, located on LG 1, 3, 7, and 8. The strong association peak on LG 1 overlapped with a known major CR QTL (qCR1a) and co‐localized with the *EVG* locus underlying the evergrowing peach dormancy mutation (*evg*). DAM1‐6 were identified in qCR1a conferring a nondormancy phenotype in *evg* peach (Li et al., 2019). There were three most significant QTLs associated with 2.8, 1.8, and 1.0 days bloom delay, respectively, in sour cherry (*Prunus cerasus* L.). These QTLS were also demonstrated to have additive effects on delaying blooming date for both individual and multiple QTLs (Cai et al., 2018).

Next‐generation sequencing (NGS) technologies are faster and cheaper than Sanger sequencing, which is frequently used in plant studies and allows for a deeper genome variant analysis (Deschamps & Campbell, 2010; Jackson, Iwata, Lee, Schmutz, & Shoemaker, 2011). So far, genome sequencing and evolutionary genetics have provided information about the origin, evolution (Ellegren, 2014; Sedivy, Wu, & Hanzawa, 2017; Velasco, Hough, Aradhya, & Ross‐Ibarra, 2016; Wu, Terol, et al., 2018; Yu et al., 2018), and domestication (Akagi, Hanada, Yaegaki, Gradziel, & Tao, 2016; Myles et al., 2011; Qiu et al., 2015; Velasco et al., 2016; Wu, Wang, et al., 2018). The first 237M long genomic map of *P. mume* was constructed in 2012, and the actual size of the genome of *P. mume* was estimated to be about 280M. The processes of chromosome fusion and fragmentation in three Genera of *Malus*, *Fragaria*, and *Prunus* were analyzed (Zhang et al., 2012). Furthermore, Zhang et al constructed the phylogenetic tree of *P. mume* cultivars by using related species of *Prunus armeniaca* and *Prunus persica* as reference. After resequencing and genome assembly with *P. armeniaca* and *P. persica* and in conjunction with the published genomes of *P. persica* and *P. mume*, GWAS analysis identified multiple quantitative trait locus regions and found that the *MYB108* gene was associated with flower color (Zhang et al., 2018).

To identify the climatic adaptation and relationship among the domesticated *P. mume* in China, 19 cultivated *P. mume* accessions from Yunnan, Sichuan, Guizhou, Jiangsu, Zhejiang, Hunan, Guangdong, Fujian, and the Taiwan provinces, the main cultivation area of *P. mume* in China, were resequenced and their accessions were locally grown for many years to be strong representatives of the cultivated *P. mume*. Based on analyses of population genetics and the evolution of cultivated *P. mume*, and selective sweeps associated with the analysis of the blooming time of the related genes, this study aimed to propose a model to explain the relationship between the climatic adaptation and blooming time of cultivated *P. mume*.

## 2 MATERIALS AND METHODS

### 2.1 Sampling information and genome resequencing

In this present study, a total of 19 *P. mume* accessions were collected and resequenced. All the accessions were from nine provinces in China (Jiangsu: R1 and R2; Sichuan: R3, R4, and R5; Yunnan: R06 and R07; Fujian: R08 and R09; Guangdong: R10 and R11; Guizhou: R12; Taiwan: R13 and R14; Zhejiang: R15, R16 and R17; and Hunan: R18 and R19, see Table 1). These sampling areas were divided into four parts: Jiangsu and Zhejiang belonged to east China; Hunan belonged to central China; Fujian, Guangdong and Taiwan belonged to south China; and Sichuan, Yunnan, and Guizhou belonged to southeast China. Genomic DNA was extracted from leaves using the CTAB method. Paired‐end DNA libraries (TruSeq^®^ DNA Library Prep Kits; Illumina) with short inserts (~500 bp) were constructed according to the manufacturer's instructions and sequenced using the HiSeq™ ×10 platforms (Illumina).

**Table 1:** Information of the blooming time partition of 19 *Prunus mume* varieties

Blooming time	Cultivar	Cultivar no.	Region	Full‐blossom period	Weather station number and location
EARLY	Taiwandaqingmei	R13	Nantou, Taiwan	Early February	59,316 Shantou
	Taiwanyanzhimei	R14	Nantou, Taiwan	Mid‐January	59,316 Shantou
	Ruanzhidalimei	R10	Puning, Guangdong	Mid‐January	59,316 Shantou
	Shuangshuidaroumei	R11	Puning, Guangdong	Mid‐January	59,316 Shantou
	Longyanmei	R08	Yongtai, Fujian	Mid‐January	58,847 Fuzhou
	Baifenmei	R09	Zhaoan, Fujian	Early January	59,316 Shantou
MID	Yunnanyanmei	R06	Lijiang, Yunnan	Mid‐January	56,651 Lijiang
	Yunnanzhaoshuimei	R07	Lijiang, Yunnan	Mid‐January	56,651 Lijiang
	Guizhousuanmei	R12	Libo, Guizhou	Early February	59,023 Hechi
	Tonglvmei	R18	Changsha, Hunan	Late February	57,662 Changde
	Xianmimei	R19	Changsha, Hunan	Late February	57,662 Changde
LATE	Sichuanqingmei	R03	Dayi, Sichuan	Late February	56,394 Chengdu
	Sichuanbaimei	R04	Dayi, Sichuan	Late February	56,394 Chengdu
	Sichuanhuangmei	R05	Dayi, Sichuan	Late February	56,394 Chengdu
	Nanhongmei	R01	Nanjing, Jiangsu	Early March	58,238 Nanjing
	Hongguangmei	R02	Nanjing, Jiangsu	Early March	58,238 Nanjing
	Ruantiaohongmei	R15	Chaoshan, Zhejiang	Mid‐March	58,454 Hangzhou
	Xiaoyezhugan	R16	Chaoshan, Zhejiang	Early March	58,454 Hangzhou
	Qingjia No.2	R17	Chaoshan, Zhejiang	Mid‐March	58,454 Hangzhou

### 2.2 Investigation of the blooming time of *Prunus mume* accessions

Because of the local climatic conditions, such as temperature and humidity are very important factors for the blooming time and the chilling hours is one of the most important conditions for the adaptation of *P. mume*. According to the phenological period of each accession and the temperature data of each meteorological station over the past 60 years from the National Centers for Environmental Information (NCEI; gis.ncdc.noaa.gov), we roughly investigate the blooming time of these 19 varieties of *P. mume* used in this study.

### 2.3 Chloroplast genome resequencing

Chloroplast DNA was extracted from the fresh leaves of each accession using a modified CTAB method. A DNA concentration >50 ng/µl was measured using a NanoDrop spectrophotometer and fragmented through sonication. Then, fragmented DNA was purified and end‐repaired, and the size was determined through gel electrophoresis. The PCR products were constructed as short‐insert (300 bp) libraries using Illumina Nextera XT and then sequenced using the HiSeq ×10 platform (Illumina). Raw reads were filtered using NGSQC Tool kit v2.3.3 to obtain high‐quality reads, and then, the chloroplast genomes were assembled by NOVOPlasty using clean data and annotated with CpGAVAS (Hu et al., 2016). The chloroplast genome of *P. persica* (NCBI Accession number NC_014697.1) was used as the reference genome in this study. The MIcroSAtellite identification tool (MISA) was used to analyze the SSR (simple sequence repeat) from the chloroplast genome.

### 2.4 Genome mapping and variant calling

Bwa software (Li & Durbin, 2009) was used to align the reads obtained from resequencing onto the *P. mume* reference genome (Zhang et al., 2012), and subsequent mutation analysis was performed. Based on the position of clean reads on the reference genome, the sequencing depth, genomic coverage, and other information from each sample were measured and the mutation was detected. Single nucleotide polymorphism (SNP) detection was primarily implemented using GATK (McKenna et al., 2010). Based on the location results of clean reads in the reference genome, using SAMtools (Li et al., 2009) to mark duplicates, GATK was used for local realignment, base recalibration, and other pretreatments to ensure the detection of SNP accuracy and then use GATK for SNP and Indel detection, filtering, and obtaining the final SNP loci. SNP and small Indel annotation were performed using SnpEff (Cingolani et al., 2012), which predicted the effects of mutations. The structural variation (SV) was detected by BreakDancer (Fan, Abbott, Larson, & Chen, 2014). There are 5 types of structural variations: deletion (DEL), insertion (INS), and inversion (INV) intra chromosomal translocation (ITX), and interchromosomal translocation (CTX). The results of the various types of detected mutations are shown in the circos diagram, using the circos software, http://circos.ca/. All the variant genes were annotated using BLASTx in the nonredundant protein (NR), Swiss‐Prot, Clusters of Orthologous Groups (COG), Gene Ontology (GO) database, and Kyoto Encyclopedia of Genes and Genomes (KEGG), and these annotations were performed according to the method of previous reports (Conesa & Gotz, 2008). The following population genetic summary statistics were then calculated for each dataset: nucleotide diversity (π; Tajima, 1989), heterozygosity (Hetobs), the inbreeding coefficient (*F*_IS_), and differentiation, as given by *F*_ST_ (Zhang et al., 2018). Nucleotide diversity is related to expected heterozygosity and is an overall measure of genetic variation.

### 2.5 Evolution analysis

To acquire more intuitive information of the evolution analysis, a principal components analysis (PCA) was performed using GCTA software, and the main clustering element was obtained. Correlation coefficients (*r* ^2^) of the alleles of the 19 varieties were calculated using PLINK software with a pair‐wise algorithm to measure the linkage disequilibrium (LD) value.

### 2.6 Population genetics analysis, identification of selective sweeps, and gene flow analysis

ADMIXTURE was used to infer population structure among the 19 samples, based on the SNPs. The population groups (*K*) varied from 2 to 10. Based on the SNPs of 19 accessions, using the peach as an exogenous reference species, a phylogenetic tree was constructed and PCA analysis was performed. The phylogenetic analysis was performed using FastTree software with the neighbor‐joining method.

To identify the genomic regions that may have been subjected to selection during domestication, we calculated nucleotide diversity (π), genetic differentiation (*F*_ST_), and the Watterson estimator (*θ*_π_) using a sliding‐window approach (50‐kb windows sliding in 10‐kb steps) with SNPs of the whole genome. The log2*θ*_π_ratio was calculated as the nucleotide diversity values for the cultivars with late‐blooming and early blooming time. The regions with significantly late *F*_ST_ values (in the 5% right tail of the empirical distribution of *F*_ST_ values) and a significant reduction in diversity (in the 5% right tail of the empirical distribution of the log2*θ*_π_ratio) were considered candidate selective sweeps.

Genes in the candidate selective sweeps were grouped into 1‐Mb nonoverlapping segments throughout the genome, according to their physical locations. Adjacent segments and segments with no more than 1‐Mb were separated and merged, while segments containing no more than five genes were discarded. GO term enrichment analyses were conducted for the genes in each merged segment along the genome using GO::TermFinder.

To estimate the gene flow between the high, mid, and early blooming time of *P. mume*, we used Treemix version 1.13 to investigate the gene flow between groups/subgroups.

### 2.7 Blooming time‐related genes analysis

Phenotypic data of blooming time were used for the association study. For the grouping of blooming time (Table 1), “low,” “mid,” and “high” groups were used. The final set of SNPs were filtered to keep those with the missing rate ≤10%, MAF ≥5%, SNP calling rate per accession ≥80%, and Hardy–Weinberg equilibrium test *p* value ≤.001. The resulting SNPs were used to perform GWAS for the blooming time using a linear mixed model (LMM) implemented in the FaST‐LMM program (Martinez et al., 2018). Raw *p*‐values were adjusted for multiple testing using the Benjamini–Hochberg procedure (Benjamini & Hochberg, 1997), and a significant association was based on a false discovery rate threshold of 0.01.

## 3 RESULTS

### 3.1 Blooming time and climate warming in the cultivated area of *Prunus mume*

Based on flowering time and the hours at 0–7.2°C, we divided the 19 varieties into three groups of blooming time: early, mid, and late (Table 1). The data for hours at 0–7.2°C in R01 and R02 were from the observation stations of Nanjing; R03, R04, and R05 were from Chengdu; R06 and R07 were from Lijiang; R08 and R09 were from Fuzhou; R10, R11, R13, and R14 were from Shantou; R12 was from Libo; R15, R16, and R17 were from Hangzhou; and R18 and R19 were from Changde (Table 1).

### 3.2 Genome, chloroplast genome resequencing, and genetic analysis of *Prunus mume*

Genomes of the 19 cultivated *P. mume* accessions were resequenced using Illumina HiSeq ×10. A total of 239.33 GB of data were obtained, and the Q30 was over 82.24%, with an average mapping rate of 93.9% (Additional file: Tables S1 and S2). The average depth of coverage was 34X–76X, and the genome coverage was 87.86% (at least one base was covered; Additional file: Table S3).

A total of 38,321,207 SNPs were identified (Additional file: Table S4). A total of 388,134 SNPs were located within the coding regions of the gene (CDs). We also identified 71,265 INDEL and 36,659 SV in the CDs (Table 2). They are filtered to only those that have a potential functional impact.

**Table 2:** Classification and statistics of nucleotide diversity produced by various mutations

Cultivar no.	SNP	INDEL	SV	Total affected genes
R01	18,343	3,255	0	18,578
R02	19,226	3,498	1,772	19,606
R15	20,616	3,674	3,864	21,045
R16	20,709	3,717	3,243	21,092
R17	20,739	3,729	3,839	21,150
R18	20,754	3,892	1,539	21,044
R19	20,713	3,859	1,580	20,957
R08	20,631	3,779	1,723	20,932
R09	20,966	3,950	1,645	21,222
R10	20,741	3,821	1,734	21,014
R11	20,710	3,818	1,761	21,005
R13	20,578	3,733	1,733	20,870
R14	20,790	3,894	1,835	21,063
R03	20,842	3,968	1,719	21,107
R04	20,514	3,759	1,792	20,819
R05	20,556	3,790	1,768	20,856
R06	20,682	3,844	1,814	20,980
R07	20,625	3,806	1,681	20,897
R12	19,399	3,479	1,617	19,739

Abbreviations: SNP, single nucleotide polymorphism; SV, structural variation.

The average number of nucleotide diversities produced by these mutations was 20,739 among these 19 cultivated accessions (Table 2), and the summary of genetic diversity in *P. mume* cultivars is shown in Table 3.

**Table 3:** Summary of genetic diversity in *Prunus mume* cultivars

Population	Sites	Polymorphic	Invariant	HetObs	π	*F*_IS_
R01	3,757,493	766,270	2,991,223	0.203931	0.101966	−1
R02	3,744,896	990,688	2,754,208	0.264544	0.132272	−1
R03	3,707,982	969,817	2,738,165	0.261548	0.130774	−1
R04	3,696,447	710,257	2,986,190	0.192146	0.096073	−1
R05	3,696,432	752,846	2,943,586	0.203668	0.101834	−1
R06	3,702,222	835,330	2,866,892	0.225629	0.112815	−1
R07	3,703,072	817,899	2,885,173	0.22087	0.110435	−1
R08	3,695,026	772,295	2,922,731	0.209009	0.104505	−1
R09	3,724,660	999,430	2,725,230	0.268328	0.134164	−1
R10	3,689,825	815,105	2,874,720	0.220906	0.110453	−1
R11	3,696,730	810,340	2,886,390	0.219205	0.109602	−1
R12	3,717,058	779,507	2,937,551	0.209711	0.104855	−1
R13	3,684,153	719,034	2,965,119	0.195169	0.097585	−1
R14	3,709,958	916,329	2,793,629	0.246992	0.123496	−1
R15	3,580,820	717,627	2,863,193	0.200409	0.100204	−1
R16	3,581,599	735,908	2,845,691	0.205469	0.102735	−1
R17	3,625,457	833,616	2,791,841	0.229934	0.114967	−1
R18	3,688,694	840,950	2,847,744	0.22798	0.11399	−1
R19	3,678,202	818,311	2,859,891	0.222476	0.111238	−1

Abbreviations: *F*_IS_, inbreeding coefficient; Hetobs, heterozygosity; π, nucleotide diversity.

A summary of the population genetic statistics is presented in Table 3. The lowest observed heterozygosity (Hetobs) in the accessions was 0.192 (Sichuan cultivar: R04), and the highest Hetobs was 0.268 (Fujian cultivar: R09). The genetic diversity (π) of cultivated *P. mume* was estimated to range from 0.096 to 0.134 (Table 3).

### 3.3 Population phylogenetic analysis of *Prunus mume* cultivars

**Figure 1:**
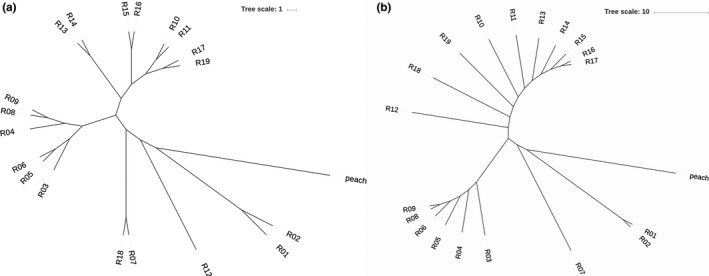
Phylogenetic trees of 19 *Prunus mume* cultivars: (a) Genome phylogenetic tree; (b) chloroplast phylogenetic tree

A subset of 388,134 SNPs was screened in greater detail to construct a neighbor‐joining tree, using the peach genome as an outgroup. The 19 cultivars were clustered into two independent clades after branching from the peach (Figure 1a). The red cluster contained four cultivars with late‐blooming time (Jiangsu and Sichuan cultivars), four cultivars with mid‐blooming time (Yunnan, Guizhou and one cultivar in Hunan), and two cultivars with early blooming time (Fujian cultivars). For the green cluster, there were four cultivars with early blooming time (Taiwan and Guangdong cultivars), one Hunan cultivar with a mid‐blooming time, and three cultivars with late‐blooming time (Zhejiang cultivars).

To get a better understanding of the phylogenetic relationships of *P. mume*, we assembled and analyzed the chloroplast genome of *P. mume*. Chloroplast genome resequencing of the 19 accessions was completed, and 239.3 GB of clean data was obtained, in which the percentage of the Q30 base was more than 82.24%. The chloroplast assembly results from 17 samples were very good, and they were able to produce a full‐circle figure with zero gaps. In addition, two samples of R16 and R17 could not be assembled into a circle, but could form better scaffolds (Additional file: Figure S1). Furthermore, the phylogenetic tree of the chloroplast showed the same cluster pattern as the genome phylogenetic tree (Figure 1b).

**Figure 2:**
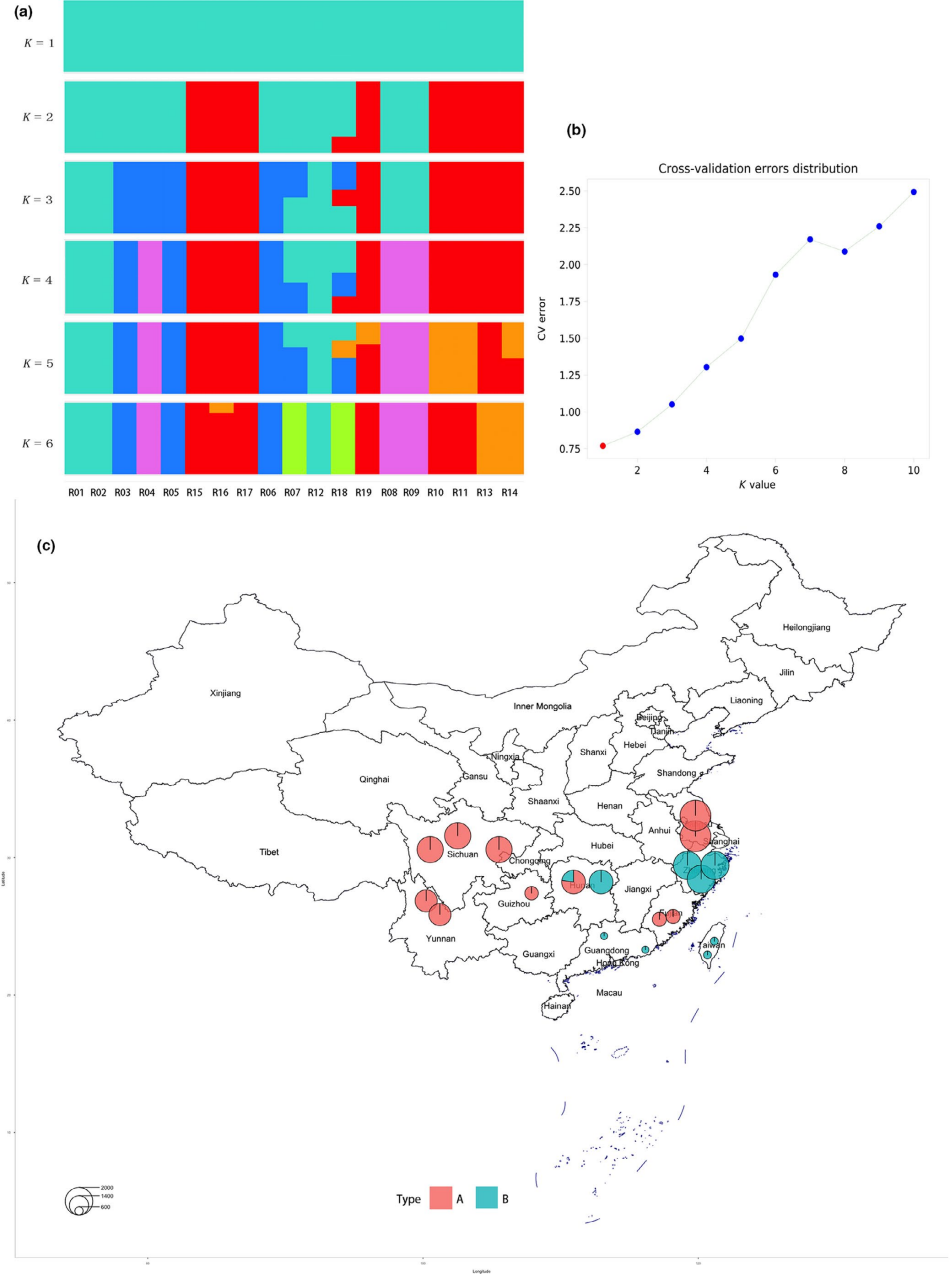
Population structure of cultivated *Prunus mume* accessions. (a) ADMIXTURE clustering analysis using a high‐density dataset. *K* = 1–6 clusters are shown. (b) The estimated prediction error was minimum on a grid of *K* = 2, which suggests that it is the most suitable *K*. (c) The admixed genetic component and geographic distribution of *P. mume* accessions (*K* = 2). The size of the circle represents the blooming time, where a small circle represents earlier blooming time, and a bigger circle represents later blooming time

We used the program ADMIXTURE to fit a model of admixture, in which an individual's genome is composed of sites from up to *K* ancestral populations. We explored *K* = 1 through six ancestral populations (Figure 2) to investigate how assumptions regarding *K* impact the inference of population structure. Assuming a *K* = 2 admixture model, population admixture patterns were driven by geographic location (Zhejiang: R15, R16 and R17; Hunan: R19; Guangdong: R10 and R11, and Taiwan: R13 and R14; Figure 2a, top panel). However, higher Ks showed substantial substructure in all three blooming time populations. Log likelihoods for successively increasing the levels of K continued to increase substantially as K increased (Figure 2b), which was not unexpected since higher values of K add more parameters to the model, thereby improving the fit.

**Figure 3:**
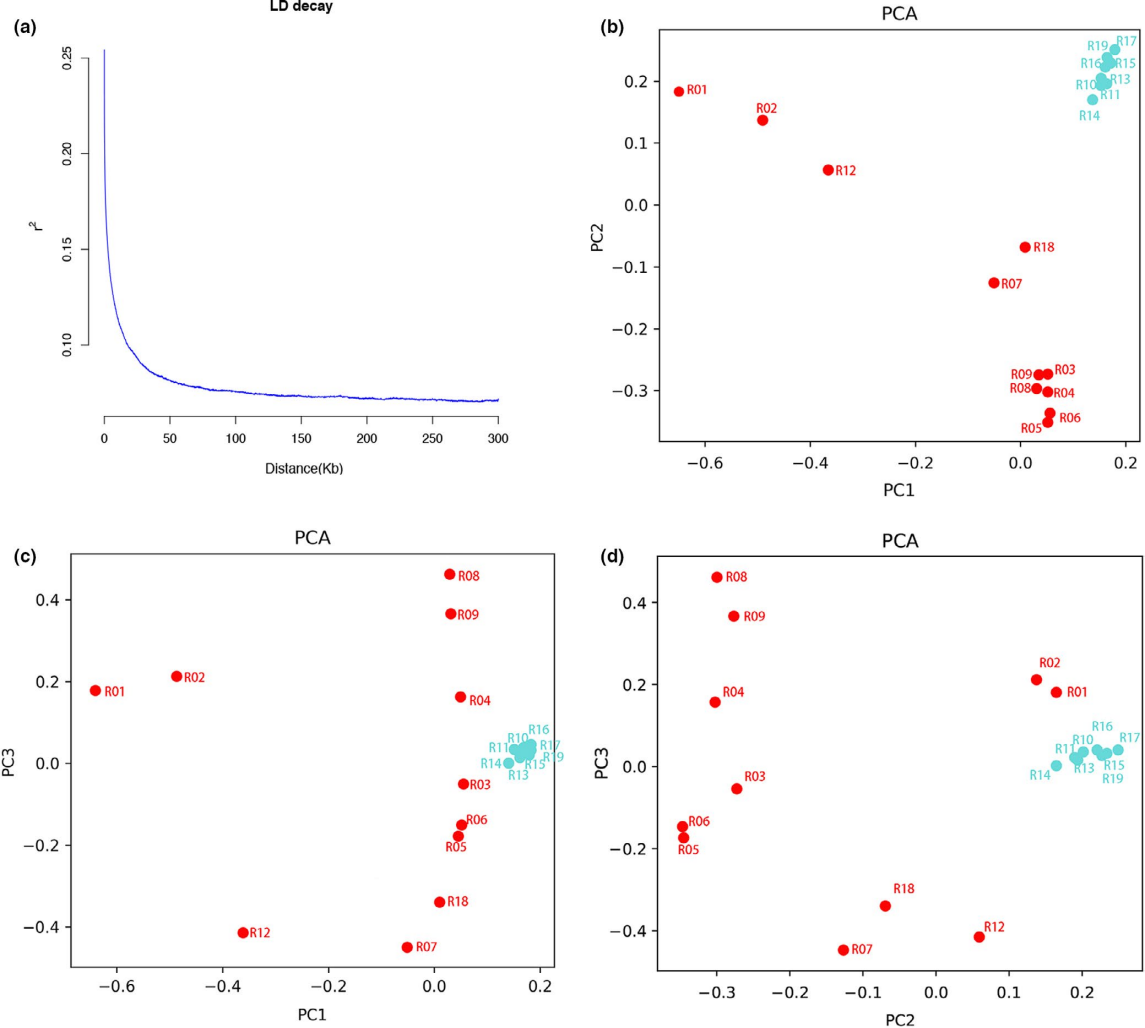
Decay of (a) linkage disequilibrium (LD) and (b, c, d) principal component analysis (PCA)

Linkage disequilibrium (LD) analysis showed that genomes have relatively short LD distances and relatively rapid LD decays (Figure 3a). The average *r* ^2^ value among *P. mume* SNPs, corresponding to the LD levels of the population, was relatively low. The average distance over which LD decayed to ~50% of its maximum value in cultivated *P. mume* was very short. Figure 3b–d showed the distribution of each accession in PCA space.

### 3.4 Natural selection of *Prunus mume* in the process of domestication

**Figure 4:**
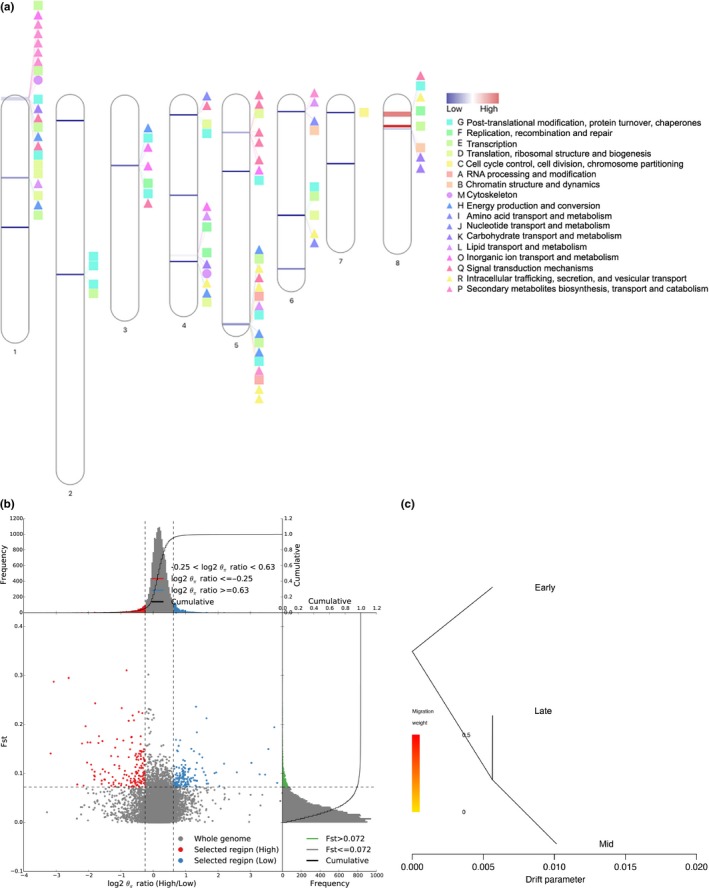
Selective sweeps and gene flow. (a) Selective sweeps and QTLs related to transcription, translation, post‐translational modification, and secondary metabolism in *Prunus mume*. The lines of each linkage group indicate selective sweeps; the colorful box, triangle and circle of each linkage group indicate QTLs. A total of 21 selective sweeps in early blooming time cultivars showed coincidence with QTLs related to transcription, translation, post‐translational modification, and secondary metabolism. The numbering at the top of the chromosome is LG numbering in Dirlewanger' paper (Dirlewanger et al., 1998), the numbering below the chromosome is LG numbering in Zhang's paper (Zhang et al., 2012). (b) Distribution of log2*θ*_π_ratio (High/Low) and *F*_st_ of 50‐kb windows with 10‐kb steps. (c) Detection of gene flow among early, mid, and late‐blooming time *Prunus mume* cultivars

To further study the genes in the transfer process, we performed selective clearance analysis. Because blooming time between the EARLY and LATE groups was significantly different, we used the EARLY and LATE‐blooming time groups to perform the selective sweep analysis (Figure 4a). Twenty‐one selective sweep regions and a total of 283 genes had been found in 19 selective sweep regions (Table S5). Considering the climate warming, the remarkable role of blooming time in distinguishing *P. mume* cultivars, genomic regions that were dramatically affected by selection during *P. mume* domestication were identified between the late‐blooming and early blooming time cultivars. The selected regions of early blooming time cultivars had a mean size of 173.8 kb, covered a total length of 3.65 Mb (1.3% of the genome; Table 4, and harbored 371 genes (Figure 4b, Table S5). The identified selective sweeps were enriched with genes associated with post‐translational modification, protein turnover, chaperones, replication, recombination and repair, transcription, translation, ribosomal structure and biogenesis, cell cycle control, cell division, chromosome partitioning, RNA processing and modification, chromatin structure and dynamics, cytoskeleton, energy production and conversion, amino acid transport and metabolism, nucleotide transport and metabolism, carbohydrate transport and metabolism, lipid transport and metabolism, inorganic ion transport and metabolism, signal transduction mechanisms, intracellular trafficking, secretion and vesicular transport, secondary metabolites biosynthesis, transport, and catabolism (Figure 4a). These biological processes are usually involved in natural selection during the process of domestication. According to the gene flow analysis, no gene flow occurred between the late and early cultivars (Figure 4c).

**Table 4:** The location of the selective sweep regions between the groups with early and late‐blooming time

Chromosomes	Starting site	Ending site	Region length (bp)
LG1 (LG6)	220,001	600,000	380,000
LG1 (LG6)	14,220,001	14,330,000	110,000
LG1 (LG6)	8,850,001	9,010,000	160,000
LG2 (LG1)	19,350,001	19,480,000	130,000
LG2 (LG1)	2,760,001	2,880,000	120,000
LG3 (LG4)	7,500,001	7,670,000	170,000
LG4 (LG3)	17,930,001	18,040,000	110,000
LG4 (LG3)	10,790,001	10,930,000	140,000
LG4 (LG3)	17,240,001	17,430,000	190,000
LG4 (LG3)	2,150,001	2,250,000	100,000
LG5 (LG2)	24,700,001	24,910,000	210,000
LG5 (LG2)	4,060,001	4,220,000	160,000
LG5 (LG2)	8,300,001	8,400,000	100,000
LG6 (LG8)	18,730,001	18,880,000	150,000
LG6 (LG8)	12,980,001	13,120,000	140,000
LG6 (LG8)	1,840,001	1,940,000	100,000
LG7 (LG5)	7,390,001	7,530,000	140,000
LG7 (LG5)	1,900,001	2,000,000	100,000
LG8 (LG7)	3,670,001	3,840,000	170,000
LG8 (LG7)	3,340,001	3,570,000	230,000
LG8 (LG7)	1,900,001	2,440,000	540,000

Note

LG numberings in bracket indicate the LG numberings in the Dirlewanger's paper.

### 3.5 Genes related to blooming time for the climatic adaptation of cultivated *Prunus mume*

**Figure 5:**
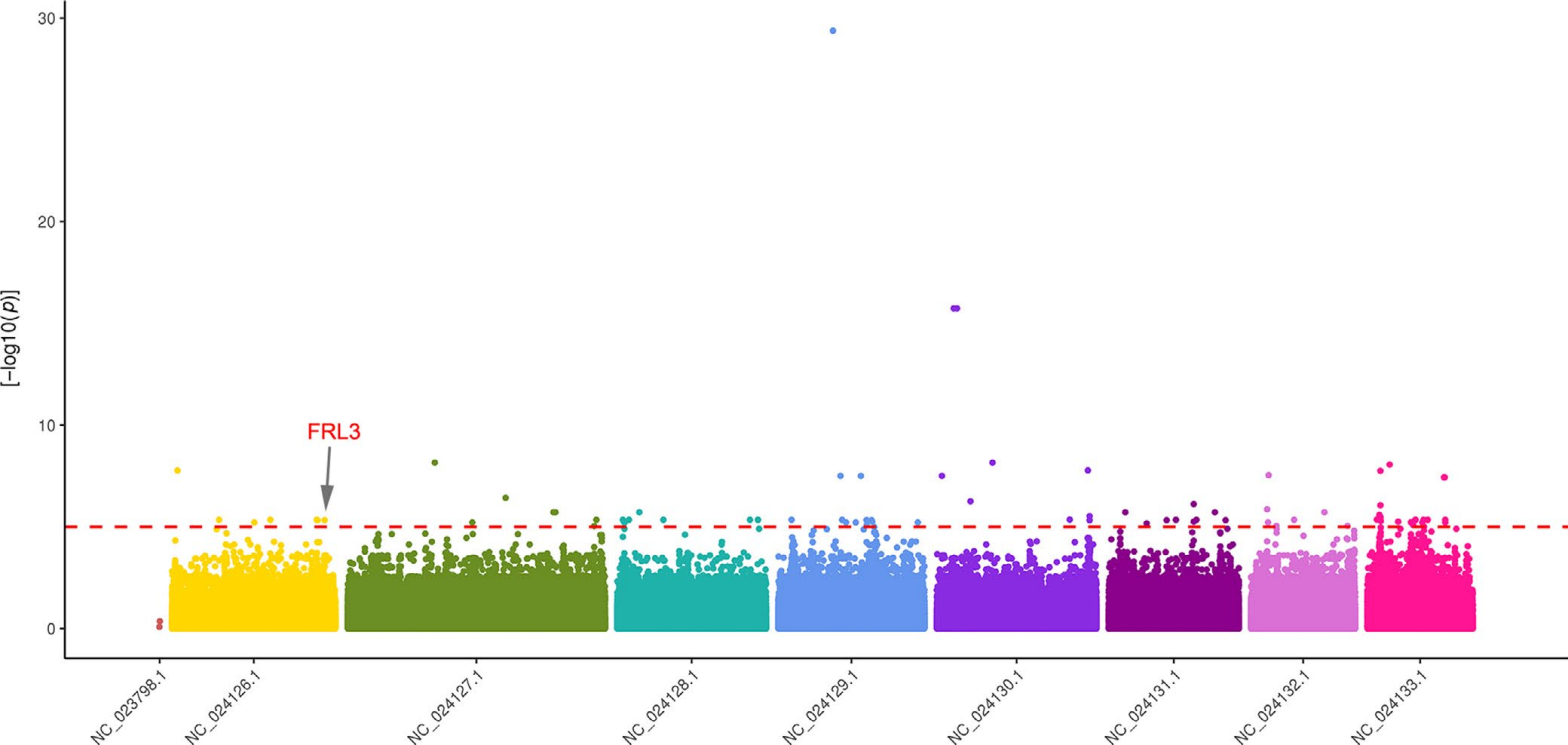
An illustration of a Manhattan plot depicting several single nucleotide polymorphisms that were strongly associated with blooming time

**Figure 6:**
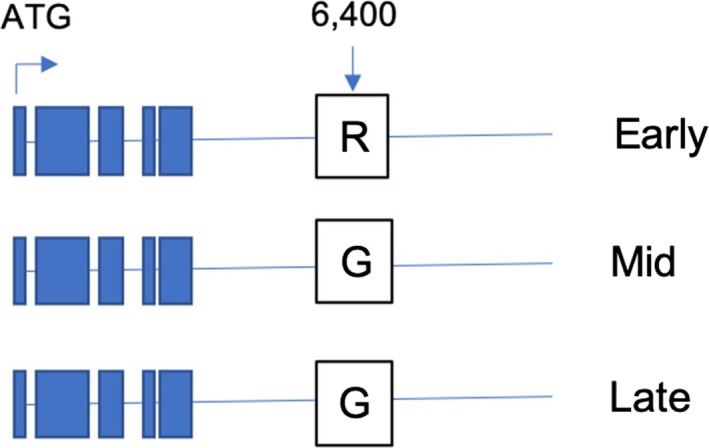
Schematic illustration of the DNA regions in *FRL3* alleles. The single nucleotide polymorphisms located at the FRL3 3′ UTR (6,400 bp) are indicated white boxes. Blue boxes represent *FRL3* gene body. Early, mid, and late represent three types of blooming time

According to the blooming time groups, we identified a total of 127 SNPs, and 54 genes associated with these SNPs (SNPs within the gene or 2 kb upstream or downstream of the gene; Figure 5, Table S6). As it turns out, our method provided quite satisfactory results about GWAS, as shown by the *p*‐value of the Q‐Q plot (Figure S2), in which the postcorrection *p*‐values were found to be close to the expected curves. Through further analysis, *FRL3* encoded FRIGIDA‐like protein 3, involved in cell differentiation and flowering, and was found on the LG1 (LG6 in Dirlewanger' paper). The relationship between SNPs related to *FRL3* and blooming time in 19 cultivars was shown in the Figure 6.

## 4 DISCUSSION

### 4.1 High genetic diversity in the fruiting of *Prunus mume*

*Prunus* is a large genus in the Rosaceae family, with more than 430 species, including multiple domesticated crops such as the almond (*Prunus dulcis* L.), apricot (*P. armeniaca* L.), cherry (*Prunus avium* L.), peach (*P. persica* L.), plum (*Prunus domestica* L.), and *P. mume*. Nucleotide diversity is related to expected heterozygosity and is an overall measure of genetic variation. *F*_ST_ represents the amount of inbreeding due to random mating in a finite population and is used as a measure of population subdivision and genetic drift.

In this study, the lowest observed heterozygosity (Hetobs) in *P. mume* accessions was 0.192 (Sichuan cultivar: R04) and the highest was 0.268 (Fujian cultivar: R09), which is higher than the genetic diversity in plants and animals (Garcia‐Elfring, 2017). The genetic diversity of cultivated *P. mume* was estimated to range from 0.096 to 0.134. Zhang et al. resequenced 333 representative *P. mume* landraces and noted that their genetic diversity was 2.01 × 10^−3^ (Zhang et al., 2018), which is lower than that of our data. The main reason for this difference may be that the accessions Zhang et al. used were ornamental *P. mume*, but our accessions were fruiting *P. mume*. It was also indicated that natural selection played an essential role during the process of the domestication of fruiting *P. mume*, but for the ornamental *P. mume*, artificial selection might have been the main factor during domestication. Many ancient books in China also record the ornamental *P. mume* cultivars and planting modes, but there was scarcely any reference record of the planting modes of fruiting *P. mume*.

For peaches, a close relative of *P. mume*, Akagi et al. analyzed modern fruit (F) and modern ornamental (O‐A) cultivars, finding a significant reduction in genetic diversity of the O‐A cluster (Akagi et al., 2016). Compared with the closely related wild species *Prunus kansuensis*, *Prunus mira*, and *Prunus davidiana*, the peach has low levels of genetic diversity (International Peach Genome Initiative et al., 2013). For the domesticated almond, it retains more genetic diversity than any of the peach species. Many woody perennial crop species, including almonds (Velasco et al., 2016), grapes (Myles et al., 2011), and apples (Gross, Henk, Richards, Fazio, & Volk, 2014), lack domestication bottlenecks, but maintain much of their ancestral genetic diversity (Velasco et al., 2016). Moreover, in this study, the analysis of selective sweep regions and gene flow also indicated that the fruiting *P. mume* barely experienced artificial selection. Altogether, this suggests that fruiting *P. mume* has high genetic diversity, with less domestication and artificial selection.

### 4.2 Effects of a warming climate on the distribution of cultivated *Prunus mume*

Kitamura et al. (2018) localized the significant QTLs controlling the leaf bud blooming time to a region on linkage group 4 (equal to LG3 in our study); they thought that this locus controlled dormancy release in *P. mume* leaf buds (Kitamura et al., 2018). In our study, the sweep selected regions in LG3 involved energy production and conversion, post‐translational modification, protein turnover, chaperones, inorganic ion transport and metabolism, replication, recombination and repair, and signal transduction metabolism. Most genes in this region are structural genes, and they might be involved in the blooming time or other processes related to blooming time. There is a receptor gene in the selective sweep regions, ultraviolet‐B receptor UVR8, that may receive environmental signals (Fernandez, Tossi, Lamattina, & Cassia, 2016). The UV‐B‐specific signaling component acts as a UV‐B photoreceptor and plays a key role in establishing UV‐protective responses in plants. When introducing a variety to a new region, the light factor must be considered, as the light intensity and light quality may be different between regions (Vanhaelewyn et al., 2016). To survive, the plant must adapt to the new environment. After receiving signals from environment, some transcription factors are involved in this adaptation, like TGA2, which is a SA‐responsive and NPR1‐dependent transcription activator (Fan & Dong, 2002); transcription factor MYB36‐like orchestrates Casparian strip formation to offer the potential for improved water and nutrient use efficiencies and enhanced resistance to abiotic stresses (Kamiya et al., 2015). Transcription factor‐like protein DPB is involved in the regulation of the G1/S transition and increases the DNA binding activity of E2F proteins after hetero‐dimerization (Kosugi & Ohashi, 2002). Transcription factor Pur‐alpha 1 specifically binds the purine‐rich double‐stranded telomeric repeated sequence 5′‐AAACCCTAA‐3′ found in promoter telo boxes (Tremousaygue, Manevski, Bardet, Lescure, & Lescure, 2010) and the ethylene‐responsive transcription factor RAP2‐12. Finally, mostly the protein and enzyme genes, such as photosystem II core complex proteins psbY (Plochinger, Schwenkert, Sydow, Schroder, & Meurer, 2016), protein phytochrome kinase substrate 4 (PKS4) (Fankhauser & Christie, 2015), and light‐inducible protein CPRF2, participate in the light reaction (Monir & Zhu, 2018). Abscisic acid‐insensitive 5‐like protein 1 (Wang, Li, Mao, Li, & Jing, 2016), ethylene‐responsive transcription factor RAP2‐12 (Kosmacz et al., 2015), auxin‐induced protein AUX28‐like (Xie et al., 2018), and auxin‐induced protein 22D‐like (Han et al., 2017) take part in plant growth and development regulated by plant hormones. E3 ubiquitin‐protein ligase CIP8‐like (Wang et al., 2019), E3 ubiquitin‐protein ligase RNF144B (Michel, Swatek, Hospenthal, & Komander, 2017), and ERAD‐associated E3 ubiquitin‐protein ligase HRD1B‐like are associated with ubiquitination (Wang, Ye, Lencer, & Hansen, 2006). Serine/threonine‐protein kinase (Dudek et al., 1997), 1‐phosphatidylinositol‐3‐phosphate 5‐kinase FAB1B (Hirano, Sato, & behavior, 2011), G‐type lectin S‐receptor‐like serine/threonine‐protein kinase (Sun et al., 2013), phosphatidylinositol 4‐phosphate 5‐kinase α (Honda et al., 1999), and so on are all genes in the selective sweep region that might allow plants to better adapt to the environment and have potential value for future studies.

We also used GWAS to analyze the genes associated with blooming time, despite only having 19 accessions. Our method provided quite satisfactory results, as shown by the *p*‐value of the Q‐Q plot, in which the *p*‐values after correction were found to be close to the expected curves. From the GWAS results, a gene related to flowering time was identified, *FRL3*. *FRL3* is a family member of FRIGIDA (*FRI*). *FRI* and *flowering locus C* (*FLC*) are the two main genes that confer the flowering traits in the winter annual *Arabidopsis thaliana* (Arabidopsis Interactome Mapping Consortium, 2011; Burn, Smyth, Peacock, & Dennis, 1993; Clarke & Dean, 1994; Lee, Bleecker, & Amasino, 1993). The FRIGIDA superfamily contains plant proteins that are similar to the FRIGIDA protein expressed in *Arabidopsis*, containing five distinct FRI subfamilies, including FRI (I), FRL1/2 (II), FRL3 (III), FRL4/4a/4b (IV), and FRL5 (V) (Risk, Laurie, Macknight, & Day, 2010). FRL1 and FRL2 have redundant roles in promoting FLC expression in the presence of FRI (Michaels, Bezerra, & Amasino, 2004); however, the roles of the other FRI subfamilies remain unclear. FRL3 may be a flowering repressor, and delayed floral timing may be regulated by gibberellic acid in bamboo (Liu, Zhu, Lin, & Ma, 2016). So, FRL3 may be an indispensable gene in the climatic adaptation process of *P. mume*, to control flowering time and normal breeding for the local climate. Therefore, FRL3 might play an important role in adapting to different local climates.

### 4.3 A proposed model for the climatic adaptation of cultivated *Prunus mume*

To explore the phylogenetic relationships of these 19 cultivated *P. mume* varieties, we performed a phylogenetic analysis of all identified SNPs, using the peach as an outgroup (Figure 1a). In the phylogenetic trees, the 19 accessions divided into two subgroups. In the first subgroup, R01 and R02, the varieties from Jiangsu province, were grouped. These two varieties are particularly close to the outgroup (Figure 1). It also seems that the Jiangsu varieties are relatively independent of the other varieties. The second subgroup was R10, R11, R13, R14, R15, R16, R17, and R19, encompassing the varieties from Guangdong, Guizhou, Taiwan, Zhejiang, and Hunan provinces. A principle component analysis (PCA) also supported our view. The Fujian, Sichuan, and Yunnan varieties clustered together. The varieties from Hunan province, R18 and R19, were separated in the phylogenetic trees, R18 clustered with R07, which are the varieties from Guizhou province and R19 clustered with R17, which is the variety from Zhejiang province. We suspect that this is due to the geographic location of Hunan (nearly in the middle of the *P. mume* cultivated region in China), so Hunan province may have been a hub in the course of the transfer of *P. mume* that linked western and eastern China.

**Figure 7:**
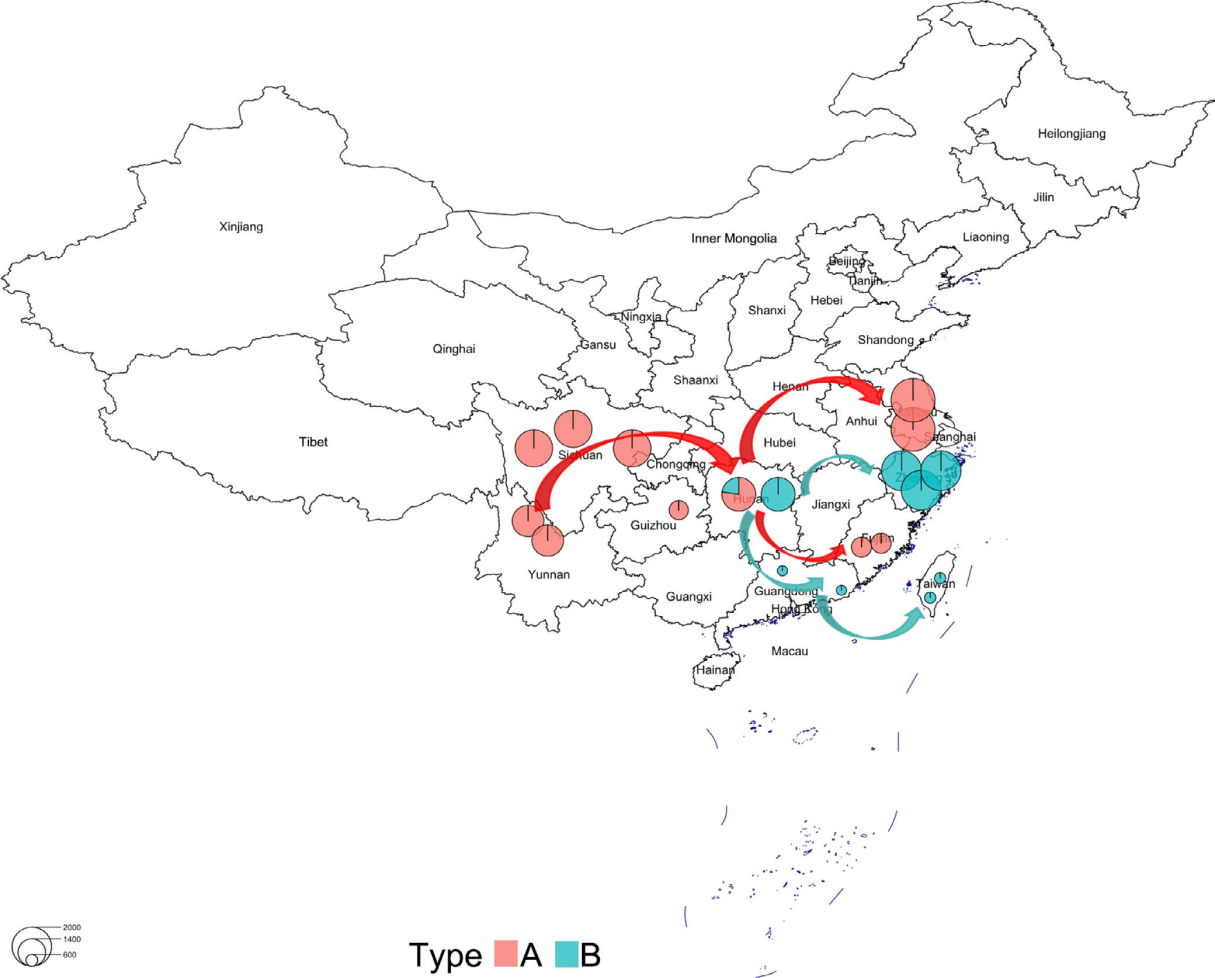
Climatic adaptation process of cultivated *Prunus mume*

After this, we used the Bayesian clustering program ADMIXTURE, changing K progressively from 2 to 10. When *K* = 2, all the cultivars separated clearly into two parts. One part included the varieties from the mainland and Fujian, while the other included the coastal region varieties. When *K* = 3, these varieties split into three parts. The cultivars of the coastal region, except for Fujian, still formed a group‐like structure similar to *K* = 2, but the mainland varieties divided into two parts: one part was a cultivar from Jiangsu and Zhejiang, and the other part was the remaining varieties. Based on the phylogenetic trees, PCA analysis, and population structure analysis, we assume that the transfer direction of cultural *P. mume* had three directions. The first direction is from Yunnan, to Sichuan, Guizhou and Hunan provinces, close to the origin area (southwest China) of *P. mume*. The second direction is a transfer to Fujian, Guangdong, and Taiwan provinces, located to the southeast from Hunan. The last direction is from Hunan to Jiangsu and Zhejiang provinces, in mid‐eastern China. In the PCA and ADMIXTURE analyses, the Sichuan and Fujian varieties were all clustered together, and Hunan province may be the hub in the transfer process of *P. mume* (Figure 7).

## 5 CONCLUSION

In this study, a genome variation mapping 19 *P. mume* accessions, collected from nine provinces in China, was performed. We found that fruiting *P. mume* has higher genetic diversity than ornamental *P. mume*. Associated with the blooming time data, 21 selective sweep regions, with a total of 283 genes involved in transcription, translation, post‐translational modification, and mostly metabolism, were identified. There was no gene flow between the late and early cultivars, which provides evidence to support a possible model of *P. mume* domestication that might include natural selection. Furthermore, we identified a total of 127 SNPs and 54 genes associated with the blooming time, and the flowering gene *FRL3*, which could affect the blooming time and the climatic adaptation of *P. mume* cultivars. This study is a major step toward understanding the climatic adaptation of *P. mume* cultivars in China and further contributes a molecular foundation for the origination and evolution of *P. mume*.

## CONFLICT OF INTEREST

None of the authors have any competing interests in the manuscript.

## AUTHOR CONTRIBUTIONS

TS, WL, HL, and XH performed the experiments. ZN prepared the materials; TS and WL wrote the manuscript; HG analyzed the bioinformatics data; SI modified the language; ZG designed the experiments.

## Data Availability

The sequence data of *P. mume* genome resequencing involved in this study are being deposited in NCBI (SRA accession is PRJNA561464). All other relevant data (Tables S5 and S6, Figures S1 and S2) supporting the findings of the study are available in this article: Figshare https://doi.org/10.6084/m9.figshare.10298780.v3.
