# Supplementary material for: Association between blooming time and climatic adaptation in Prunus mume
Source: Ecol Evol. 2019 Dec 20;10(1):292–306. doi: 10.1002/ece3.5894 (PMC6972806; doi:10.1002/ece3.5894)
Supplement: Supplementary file 1 [file ECE3-10-292-s001.docx]

Table S1 Evaluation of sequencing data

| Sample | Clean Reads | Base Number | GC Content | %≥Q30 |
| --- | --- | --- | --- | --- |
| R01 | 35,575,193 | 11,644,238,850 | 38.96 | 93.91 |
| R02 | 61,508,013 | 18,452,403,900 | 39.03 | 89.51 |
| R03 | 40,245,006 | 12,073,501,800 | 38.91 | 88.65 |
| R04 | 45,289,206 | 13,586,761,800 | 39.98 | 84.52 |
| R05 | 45,143,631 | 13,543,089,300 | 38.92 | 88.01 |
| R06 | 44,851,838 | 13,455,551,400 | 38.75 | 89.20 |
| R07 | 40,815,276 | 12,244,582,800 | 39.58 | 89.14 |
| R08 | 47,564,833 | 14,269,449,900 | 39.50 | 84.74 |
| R09 | 45,526,170 | 13,657,851,000 | 38.80 | 88.72 |
| R10 | 42,400,023 | 12,720,006,900 | 39.10 | 88.98 |
| R11 | 40,212,552 | 12,063,765,600 | 38.93 | 86.34 |
| R12 | 42,053,066 | 11,644,238,850 | 39.35 | 82.24 |
| R13 | 46,437,573 | 13,931,271,900 | 38.99 | 85.73 |
| R14 | 46,113,950 | 13,834,185,000 | 39.12 | 86.85 |
| R15 | 42,860,525 | 8,572,105,000 | 40.82 | 91.05 |
| R16 | 39,743,375 | 11,923,012,500 | 43.62 | 91.43 |
| R17 | 44,618,139 | 8,923,627,800 | 39.78 | 90.32 |
| R18 | 37,383,716 | 11,215,114,800 | 39.44 | 93.23 |
| R19 | 38,598,292 | 11,579,487,600 | 39.47 | 93.63 |

The column of “Sample” is the serial number of 19 varieties. The column of “Clean Reads” is the sum of pair-end Reads in the clean data of 19 varieties. The column of “Base Number” is total base number of Clean Data. The column of “GC Content” is the GC Content of each variety. The column of “%≥Q30” is the percentage of bases with a mass value greater than or equal to 30.

Table S2 Mapping result compared to the reference genome of Prunus mume.

| sample | number of reads | number of mapped reads | duplication rate | mean mapping quality |
| --- | --- | --- | --- | --- |
| R01 | 72,431,645 | 98.84% | 29.33% | 52.2015 |
| R02 | 125,176,290 | 99.14% | 35.28% | 51.625 |
| R03 | 82,096,169 | 98.85% | 28.38% | 50.6651 |
| R04 | 92,645,812 | 98.41% | 30.46% | 50.3746 |
| R05 | 92,107,374 | 96.01% | 30.49% | 50.5304 |
| R06 | 91,477,516 | 98.35% | 29.59% | 50.573 |
| R07 | 83,296,117 | 93.90% | 29.05% | 50.7503 |
| R08 | 97,103,175 | 98.62% | 31.52% | 50.4666 |
| R09 | 92,893,347 | 98.94% | 29.08% | 50.6566 |
| R10 | 86,538,217 | 98.84% | 30.09% | 50.5352 |
| R11 | 82,006,812 | 99.09% | 29.67% | 50.5681 |
| R12 | 85,581,977 | 99.10% | 30.26% | 51.3216 |
| R13 | 94,721,436 | 98.78% | 31.03% | 50.4517 |
| R14 | 93,996,443 | 98.99% | 30.70% | 50.5987 |
| R15 | 86,269,602 | 97.20% | 25.71% | 49.2682 |
| R16 | 82,583,791 | 95.67% | 51.73% | 50.2478 |
| R17 | 89,832,778 | 97.45% | 26.89% | 49.2913 |
| R18 | 76,244,362 | 99.24% | 37.36% | 50.8411 |
| R19 | 78,798,257 | 99.07% | 39.69% | 50.7944 |

Note: number of reads is the total number of reads that located in the reference genome. Rate of mapped reads is the number of Clean Reads localized to the reference genome accounted for a percentage of all Clean Reads. Duplication rate is the percentage which cause by the fragment duplication which due to PCR. Mean mapping quality is the average quality of all reads located on the reference genome.

Table S3 Sequencing depth and coverage analysis of accessions

| sample | Ave_depth | Cov_ratio_1X(%) | Cov_ratio_5X(%) | Cov_ratio_10X(%) |
| --- | --- | --- | --- | --- |
| R01 | 44.0246 | 91.05 | 89.4 | 87.39 |
| R02 | 76.4917 | 90.4 | 89.1 | 88.09 |
| R03 | 49.7271 | 88.85 | 86.66 | 84.87 |
| R04 | 54.9672 | 88.94 | 86.65 | 85.05 |
| R05 | 54.0128 | 88.68 | 86.52 | 84.87 |
| R06 | 55.245 | 88.81 | 86.67 | 85.04 |
| R07 | 47.5603 | 89.04 | 86.9 | 84.97 |
| R08 | 58.235 | 88.83 | 86.69 | 85.22 |
| R09 | 56.3737 | 89.31 | 87.38 | 85.86 |
| R10 | 52.2387 | 88.39 | 86.19 | 84.48 |
| R11 | 49.759 | 88.57 | 86.3 | 84.53 |
| R12 | 52.1206 | 89.58 | 87.76 | 86.26 |
| R13 | 57.222 | 88.27 | 86.09 | 84.51 |
| R14 | 56.9485 | 88.87 | 86.75 | 85.15 |
| R15 | 34.9117 | 88.03 | 83.06 | 76.44 |
| R16 | 45.9165 | 87.86 | 83.22 | 76.94 |
| R17 | 36.4253 | 88.49 | 85.36 | 81.42 |
| R18 | 46.5725 | 88.53 | 86.33 | 84.25 |
| R19 | 47.9228 | 88.19 | 85.99 | 84.04 |

Table S4 The information of SNP Calling.

| sample | SNP number | Transition | Transversion | Ti/Tv | Heterozygosity | Homozygosity |
| --- | --- | --- | --- | --- | --- | --- |
| R01 | 1,503,215 | 966,253 | 536,962 | 1.8 | 1,151,421 | 376,537 |
| R02 | 1,750,298 | 1,128,572 | 621,726 | 1.82 | 1,241,781 | 536,526 |
| R03 | 2,188,085 | 1,418,464 | 769,621 | 1.84 | 1,130,452 | 1,089,185 |
| R04 | 2,031,818 | 1,318,212 | 713,606 | 1.85 | 836,308 | 1,226,725 |
| R05 | 2,055,395 | 1,332,468 | 722,927 | 1.84 | 871,347 | 1,215,472 |
| R06 | 2,101,586 | 1,362,696 | 738,890 | 1.84 | 957,961 | 1,174,761 |
| R07 | 2,061,165 | 1,335,805 | 725,360 | 1.84 | 1,019,767 | 1,073,842 |
| R08 | 2,066,334 | 1,340,258 | 726,076 | 1.85 | 920,314 | 1,177,283 |
| R09 | 2,222,600 | 1,440,874 | 781,726 | 1.84 | 1,191,417 | 1,062,977 |
| R10 | 2,092,020 | 1,355,918 | 736,102 | 1.84 | 934,059 | 1,189,065 |
| R11 | 2,088,136 | 1,354,323 | 733,813 | 1.85 | 928,347 | 1,191,080 |
| R12 | 1,786,811 | 1,156,186 | 630,625 | 1.83 | 1,024,070 | 792,142 |
| R13 | 2,032,752 | 1,317,430 | 715,322 | 1.84 | 827,624 | 1,235,818 |
| R14 | 2,150,348 | 1,394,822 | 755,526 | 1.85 | 1,054,155 | 1,127,174 |
| R15 | 1,951,911 | 1,267,865 | 684,046 | 1.85 | 815,263 | 1,164,789 |
| R16 | 1,968,894 | 1,279,901 | 688,993 | 1.86 | 831,861 | 1,165,312 |
| R17 | 2,051,162 | 1,330,951 | 720,211 | 1.85 | 935,075 | 1,145,712 |
| R18 | 2,131,193 | 1,381,068 | 750,125 | 1.84 | 1,086,231 | 1,077,266 |
| R19 | 2,087,484 | 1,354,336 | 733,148 | 1.85 | 933,730 | 1,184,671 |

“Transition” means the number of SNP of the transition type (A/G and T/C; Ts). “Transversion” means the number of SNP of the transversion type (A/T, A/C, T/G, C/G; Tv). “Ti/Tv” means the transition and transversion type SNP ratio. “Heterozygosity” means the number of heterozygous types of SNP. “Homozygosity” means the number of homozygous types of SNP.
