# Supplementary material for: Association between blooming time and climatic adaptation in Prunus mume
Source: Ecol Evol. 2019 Dec 20;10(1):292–306. doi: 10.1002/ece3.5894 (PMC6972806; doi:10.1002/ece3.5894)
Supplement: Supplementary file 2 [file ECE3-10-292-s002.docx]

Table S5 The gene information inside the Selective Sweep region

| Chromosome | Selective Sweep Region | Gene ID | Gene Description |
| --- | --- | --- | --- |
| LG1 | 220001~600000 | LOC103342554 | uncharacterized LOC103342554 |
|  |  | LOC103319262 | TATA-binding protein-associated factor BTAF1 |
|  |  | LOC103319355 | alpha-ketoglutarate-dependent dioxygenase alkB homolog 6 |
|  |  | LOC103319616 | L-ascorbate oxidase homolog |
|  |  | LOC103319513 | L-ascorbate oxidase homolog |
|  |  | LOC103319734 | L-ascorbate oxidase homolog |
|  |  | LOC107880819 | uncharacterized LOC107880819 |
|  |  | LOC103319834 | L-ascorbate oxidase homolog |
|  |  | LOC103319935 | transcription factor Pur-alpha 1 |
|  |  | LOC103320087 | pentatricopeptide repeat-containing protein At5g56310 |
|  |  | LOC103323967 | kinesin-like protein KIF22 |
|  |  | LOC103320246 | uncharacterized LOC103320246 |
|  |  | LOC103320338 | nuclear pore complex protein NUP214 |
|  |  | LOC103320436 | uncharacterized LOC103320436 |
|  |  | LOC103320533 | uncharacterized LOC103320533 |
|  |  | LOC103320759 | E3 ubiquitin-protein ligase CIP8-like |
|  |  | LOC103320916 | pyruvate kinase, cytosolic isozyme |
|  |  | LOC103321018 | glucosidase 2 subunit beta |
|  |  | LOC103321114 | uncharacterized LOC103321114 |
|  |  | LOC103321204 | TATA-box-binding protein |
|  |  | LOC103321304 | 2-oxoisovalerate dehydrogenase subunit beta 1, mitochondrial |
|  |  | LOC103321408 | uncharacterized LOC103321408 |
|  |  | LOC103321565 | subtilisin-like protease SBT3.18 |
|  |  | LOC103321663 | probable receptor-like protein kinase At5g56460 |
|  |  | LOC103321762 | ruBisCO large subunit-binding protein subunit beta, chloroplastic |
|  |  | LOC103321855 | pumilio homolog 12 |
|  |  | LOC103321955 | uncharacterized LOC103321955 |
|  |  | LOC103322044 | uncharacterized LOC103322044 |
|  |  | LOC103322137 | lysine--tRNA ligase, chloroplastic/mitochondria |
|  |  | LOC103322287 | uncharacterized LOC103322287 |
|  |  | LOC103322378 | uncharacterized LOC103322378 |
|  |  | LOC103322637 | uncharacterized LOC103322637 |
|  |  | LOC103322730 | calpain-type cysteine protease DEK1 |
|  |  | LOC103322820 | uncharacterized LOC103322820 |
|  |  | LOC103322914 | arabinogalactan peptide 13-like |
|  |  | LOC103323013 | mediator of RNA polymerase II transcription subunit 13 |
|  | 8850001~9010000 | LOC107881807 | 30S ribosomal protein S17-like |
|  |  | LOC103327065 | flagellar radial spoke protein 5 |
|  |  | LOC103327078 | histidine-containing phosphotransfer protein 1-like |
|  |  | LOC103327086 | uncharacterized LOC103327086 |
|  |  | LOC103327861 | probable LRR receptor-like serine/threonine-protein kinase At1g53420 |
|  |  | LOC103328110 | pectinesterase PPME1-like |
|  |  | LOC103327890 | CEN-like protein 2 |
|  |  | LOC103328121 | uncharacterized LOC103328121 |
|  |  | LOC103328131 | receptor-like protein 12 |
|  |  | LOC103328157 | uncharacterized LOC103328157 |
|  |  | LOC103327901 | uncharacterized LOC103327901 |
|  |  | LOC103327914 | uncharacterized LOC103327914 |
|  |  | LOC103327923 | uncharacterized LOC103327923 |
|  |  | LOC103327933 | ABSCISIC ACID-INSENSITIVE 5-like protein 1 |
|  |  | LOC103327943 | uncharacterized LOC103327943 |
|  |  | LOC103327958 | transcription factor TGA2 |
|  | 14220001~14330000 | LOC103331927 | protein MKS1 |
|  |  | LOC103331954 | uridylate kinase |
| LG 2 | 2760001~2880000 | LOC103319257 | protein DETOXIFICATION 49-like |
|  |  | LOC107880475 | uncharacterized LOC107880475 |
|  |  | LOC107880244 | uncharacterized LOC107880244 |
|  |  | LOC107880246 | uncharacterized LOC107880246 |
|  | 19350001~19480000 | LOC103321882 | ultraviolet-B receptor UVR8 |
|  |  | LOC103321883 | ALBINO3-like protein 1, chloroplastic |
|  |  | LOC103321884 | photosystem II core complex proteins psbY, chloroplastic |
|  |  | LOC103321885 | uncharacterized LOC103321885 |
|  |  | LOC103321935 | uncharacterized LOC103321935 |
|  |  | LOC103321886 | very-long-chain 3-oxoacyl-CoA reductase 1-like |
|  |  | LOC103321887 | very-long-chain 3-oxoacyl-CoA reductase 1-like |
|  |  | LOC103321888 | very-long-chain 3-oxoacyl-CoA reductase 1-like |
|  |  | LOC103321890 | very-long-chain 3-oxoacyl-CoA reductase-like protein At1g24470 |
|  |  | LOC103321891 | regulator of G-protein signaling 1 |
|  |  | LOC103321892 | uncharacterized LOC103321892 |
|  |  | LOC103321893 | uncharacterized LOC103321893 |
|  |  | LOC103321894 | myosin-11 |
|  |  | LOC103321895 | RING-H2 finger protein ATL47-like |
|  |  | LOC103321896 | probable LRR receptor-like serine/threonine-protein kinase At1g67720 |
|  |  | LOC103321897 | uncharacterized LOC103321897 |
|  |  | LOC103321899 | uncharacterized LOC103321899 |
|  |  | LOC103321900 | two-component response regulator ARR11 |
| LG3 | 7500001~7670000 | LOC103325562 | isocitrate dehydrogenase [NADP] |
|  |  | LOC103325564 | serine/threonine-protein kinase RHS3 |
|  |  | LOC103325565 | UPF0678 fatty acid-binding protein-like protein At1g79260 |
|  |  | LOC103325566 | aspartyl protease family protein 2 |
|  |  | LOC103325567 | YTH domain-containing family protein 2 |
|  |  | LOC103325629 | nuclear-pore anchor |
|  |  | LOC103325630 | (E,E)-alpha-farnesene synthase-like |
|  |  | LOC103325631 | (E,E)-alpha-farnesene synthase-like |
|  |  | LOC103325568 | (E,E)-alpha-farnesene synthase-like |
|  |  | LOC103325632 | (E,E)-alpha-farnesene synthase-like |
|  |  | LOC103325569 | uncharacterized LOC103325569 |
|  |  | LOC103325633 | putative calcium-transporting ATPase 13, plasma membrane-type |
|  |  | LOC107880801 | uncharacterized LOC107880801 |
| LG4 | 2150001~2250000 | LOC103327552 | pectinesterase 3 |
|  |  | LOC103327553 | alanine aminotransferase 2, mitochondrial-like |
|  |  | LOC103327555 | uncharacterized LOC103327555 |
|  |  | LOC103327556 | uncharacterized LOC103327556 |
|  |  | LOC103327557 | 1-phosphatidylinositol-3-phosphate 5-kinase FAB1B |
|  |  | LOC103327559 | protein LURP-one-related 11-like |
|  |  | LOC103327558 | translation initiation factor eIF-2B subunit alpha-like |
|  |  | LOC107880984 | protein SENESCENCE-ASSOCIATED GENE 21, mitochondrial-like |
|  |  | LOC103327560 | uncharacterized LOC103327560 |
|  |  | LOC103327562 | AT-rich interactive domain-containing protein 4 |
|  |  | LOC103327738 | E3 ubiquitin-protein ligase RNF144B |
|  |  | LOC103327740 | subtilisin-like protease SBT1.5 |
|  |  | LOC103327563 | ethylene-responsive transcription factor RAP2-12 |
|  |  | LOC103327741 | uncharacterized LOC103327741 |
|  |  | LOC103327564 | GDSL esterase/lipase 5 |
|  | 10790001~10930000 | LOC103328504 | plant cysteine oxidase 4-like |
|  |  | LOC107880913 | uncharacterized LOC107880913 |
|  |  | LOC103328515 | LOB domain-containing protein 2 |
|  | 17240001~17430000 | LOC107880928 | G-type lectin S-receptor-like serine/threonine-protein kinase At1g11410 |
|  |  | LOC103329349 | G-type lectin S-receptor-like serine/threonine-protein kinase At1g11330 |
|  |  | LOC107880929 | putative inactive G-type lectin S-receptor-like serine/threonine-protein kinase SRK |
|  |  | LOC103329284 | CRC domain-containing protein TSO1-like |
|  |  | LOC103329350 | acyl-protein thioesterase 2-like |
|  |  | LOC107880107 | uncharacterized LOC107880107 |
|  |  | LOC103329286 | uncharacterized LOC103329286 |
|  |  | LOC103329287 | probable beta-1,3-galactosyltransferase 2 |
|  |  | LOC103329288 | autophagy-related protein 8d-like |
|  |  | LOC103329354 | procyclic form-specific polypeptide B-alpha-like |
|  |  | LOC103329289 | 2-alkenal reductase (NADP(+)-dependent)-like |
|  |  | LOC107880960 | NADP-dependent alkenal double bond reductase P1-like |
|  |  | LOC103329290 | 2-alkenal reductase (NADP(+)-dependent)-like |
|  | 17930001~18040000 | LOC103329384 | aconitate hydratase, cytoplasmic |
|  |  | LOC103329443 | uncharacterized LOC103329443 |
|  |  | LOC103329386 | uncharacterized GPI-anchored protein At1g61900-like |
|  |  | LOC103329444 | protein DETOXIFICATION 40-like |
|  |  | LOC103329387 | protein DETOXIFICATION 40-like |
|  |  | LOC103329388 | glutamate receptor 3.7 |
|  |  | LOC103329389 | glutamate receptor 3.4 |
|  |  | LOC103329390 | septum-promoting GTP-binding protein 1 |
|  |  | LOC103329391 | uncharacterized LOC103329391 |
|  |  | LOC103329392 | transmembrane protein 97-like |
|  |  | LOC103329393 | transmembrane protein 97-like |
|  |  | LOC103329394 | homeobox-leucine zipper protein HDG2 |
|  |  | LOC103329395 | uncharacterized LOC103329395 |
|  |  | LOC103329447 | uncharacterized LOC103329447 |
|  |  | LOC103329396 | syntaxin-132-like |
|  |  | LOC103329397 | isocitrate lyase |
|  |  | LOC103329398 | uncharacterized LOC103329398 |
|  |  | LOC103329399 | transcription factor CPC-like |
|  |  | LOC103329400 | protein translation factor SUI1 homolog |
| LG5 | 4060001~4220000 | LOC103330865 | uncharacterized LOC103330865 |
|  |  | LOC103330840 | phosphatidylinositol 4-phosphate 5-kinase 6 |
|  |  | LOC103330842 | uncharacterized LOC103330842 |
|  |  | LOC103330867 | putative disease resistance RPP13-like protein 1 |
|  |  | LOC103330843 | 40S ribosomal protein S10-like |
|  |  | LOC103330844 | putative disease resistance RPP13-like protein 1 |
|  |  | LOC107881093 | uncharacterized LOC107881093 |
|  |  | LOC103330869 | NADPH:quinone oxidoreductase-like |
|  |  | LOC103330871 | putative disease resistance RPP13-like protein 1 |
|  |  | LOC103330845 | uncharacterized LOC103330845 |
|  |  | LOC103330846 | NADPH:quinone oxidoreductase-like |
|  |  | LOC103330847 | NADPH:quinone oxidoreductase-like |
|  |  | LOC103330872 | cation/H(+) antiporter 15-like |
|  | 8300001!8400000 | LOC103331144 | GDSL esterase/lipase At5g03610-like |
|  |  | LOC103331137 | glycine-rich RNA-binding protein 4, mitochondrial-like |
|  |  | LOC107881114 | GDSL esterase/lipase At5g03610-like |
|  |  | LOC103331146 | GDSL esterase/lipase At5g03610-like |
|  |  | LOC107881115 | uncharacterized LOC107881115 |
|  | 24700001~24910000 | LOC103333478 | V-type proton ATPase subunit H |
|  |  | LOC103333479 | uncharacterized LOC103333479 |
|  |  | LOC103333480 | WEB family protein At3g02930, chloroplastic-like |
|  |  | LOC103333481 | uncharacterized LOC103333481 |
|  |  | LOC103333482 | uncharacterized LOC103333482 |
|  |  | LOC103333484 | protein FAR1-RELATED SEQUENCE 4 |
|  |  | LOC103333485 | inositol-tetrakisphosphate 1-kinase 1-like |
|  |  | LOC103333740 | transcription factor MYB36-like |
|  |  | LOC103333486 | S-norcoclaurine synthase 1-like |
|  |  | LOC103333487 | protein EXORDIUM-like 4 |
|  |  | LOC103333488 | histone acetyltransferase MCC1 |
|  |  | LOC103333489 | syntaxin-22 |
|  |  | LOC103333490 | binding partner of ACD11 1 |
|  |  | LOC103333741 | uncharacterized protein At1g24485-lik |
|  |  | LOC103333743 | probable calcium-binding protein CML18 |
|  |  | LOC103333491 | peptidyl-tRNA hydrolase 2, mitochondrial |
|  |  | LOC103333492 | target of Myb protein 1 |
|  |  | LOC103333493 | probable arabinosyltransferase ARAD1 |
|  |  | LOC103333494 | cellulose synthase-like protein D3 |
|  |  | LOC103333744 | uncharacterized LOC103333744 |
|  |  | LOC103333495 | transducin beta-like protein 3 |
|  |  | LOC103333496 | 4-coumarate--CoA ligase 2 |
|  |  | LOC103333498 | ATPase family AAA domain-containing protein 3-B-like |
|  |  | LOC103333499 | NADH dehydrogenase [ubiquinone] iron-sulfur protein 6, mitochondrial |
|  |  | LOC103333500 | pentatricopeptide repeat-containing protein At1g77170 |
|  |  | LOC103333501 | pentatricopeptide repeat-containing protein At5g27270 |
|  |  | LOC103333502 | transcription factor-like protein DPB |
|  |  | LOC103333503 | protein SMG9-like |
|  |  | LOC103333504 | uncharacterized LOC103333504 |
|  |  | LOC103333505 | D-xylose-proton symporter-like 2 |
|  |  | LOC103333506 | probable NADH dehydrogenase [ubiquinone] 1 alpha subcomplex subunit 12 |
|  |  | LOC103333507 | ERAD-associated E3 ubiquitin-protein ligase HRD1B-like |
|  |  | LOC103333509 | laccase-15-like |
|  |  | LOC103333510 | protein STABILIZED1-like |
|  |  | LOC103333511 | ER lumen protein-retaining receptor-like |
|  |  | LOC103333512 | protein EXPORTIN 1A |
|  |  | LOC103333513 | anthocyanidin 3-O-glucosyltransferase 2-like |
|  |  | LOC103333514 | ADP-ribosylation factor 1-like 2 |
|  |  | LOC103333745 | uncharacterized LOC103333745 |
|  |  | LOC103333515 | serine/threonine-protein phosphatase 4 regulatory subunit 2-A |
|  |  | LOC103333516 | protein TPR2-like |
|  |  | LOC103333746 | serine/threonine-protein kinase TIO-like |
| LG6 | 1840001~1940000 | LOC103334247 | glutathione reductase, chloroplastic |
|  |  | LOC103334163 | U3 small nucleolar RNA-associated protein 18 homolog |
|  |  | LOC103334164 | phospholipase A1-Igamma2, chloroplastic |
|  |  | LOC103334165 | F-box/LRR-repeat protein 17 |
|  |  | LOC103334166 | putative uridine kinase C227.14 |
|  |  | LOC103334167 | auxin-induced protein AUX28-like |
|  |  | LOC103334168 | auxin-induced protein 22D-like |
|  |  | LOC103334170 | protein OSB4, chloroplastic-like |
|  |  | LOC103334171 | transcription factor UNE12 |
|  |  | LOC103334172 | tryptophan synthase alpha chain |
|  |  | LOC103334173 | light-inducible protein CPRF2 |
|  |  | LOC103334174 | histone acetyltransferase GCN5 |
|  |  | LOC103334175 | uncharacterized LOC103334175 |
|  |  | LOC103334176 | guanylate-binding protein 4 |
|  |  | LOC103334177 | uncharacterized LOC103334177 |
|  |  | LOC103334178 | histone H2A variant 1 |
|  |  | LOC103334180 | beta-carotene isomerase D27, chloroplastic |
|  |  | LOC103334181 | protein LONGIFOLIA 1 |
|  | 12980001~13120000 | LOC103335884 | guanosine nucleotide diphosphate dissociation inhibitor At5g09550 |
|  |  | LOC103335885 | MADS-box protein SVP-like |
|  |  | LOC103335886 | ribosome biogenesis protein BMS1 homolog |
|  |  | LOC103335887 | uncharacterized LOC103335887 |
|  |  | LOC103335888 | uncharacterized LOC103335888 |
|  | 18730001~18880000 | LOC103336310 | uncharacterized LOC103336310 |
|  |  | LOC103336337 | protein PHLOEM PROTEIN 2-LIKE A1-like |
|  |  | LOC103336339 | protein PHLOEM PROTEIN 2-LIKE A1-like |
|  |  | LOC107881383 | uncharacterized mitochondrial protein AtMg00810-like |
|  |  | LOC103336311 | uncharacterized LOC103336311 |
|  |  | LOC103336312 | uncharacterized LOC103336312 |
|  |  | LOC107881384 | uncharacterized mitochondrial protein AtMg00810-like |
|  |  | LOC103336341 | uncharacterized LOC103336341 |
|  |  | LOC103336313 | uncharacterized LOC103336313 |
| LG7 | 1900001~2000000 | LOC103336787 | nuclear pore complex protein NUP160 |
|  |  | LOC103336788 | MOB kinase activator-like 1A |
|  |  | LOC103336789 | transmembrane protein 45A |
|  |  | LOC103336790 | protein ENHANCED DISEASE RESISTANCE 2 |
|  |  | LOC103336791 | uncharacterized LOC103336791 |
|  | 7390001~7530000 | null | null |
| LG 8 | 1900001~2440000 | LOC103339098 | uncharacterized LOC103339098 |
|  |  | LOC103339099 | L-2-hydroxyglutarate dehydrogenase, mitochondrial |
|  |  | LOC103339100 | protein PHR1-LIKE 1-like |
|  |  | LOC103339117 | protein PHYTOCHROME KINASE SUBSTRATE 4 |
|  |  | LOC103339101 | 14 kDa zinc-binding protein |
|  |  | LOC107881746 | uncharacterized LOC107881746 |
|  |  | LOC107881673 | uncharacterized LOC107881673 |
|  |  | LOC103339102 | proteasome subunit alpha type-3 |
|  |  | LOC103339103 | uncharacterized LOC103339103 |
|  |  | LOC103339104 | probable protein S-acyltransferase 4 |
|  |  | LOC103339106 | magnesium-protoporphyrin IX monomethyl ester [oxidative] cyclase, chloroplastic |
|  |  | LOC103339107 | protein SYM1 |
|  |  | LOC103339120 | uncharacterized LOC103339120 |
|  |  | LOC103339108 | uncharacterized LOC103339108 |
|  |  | LOC103339109 | syntaxin-51-like |
|  |  | LOC103339110 | uncharacterized LOC103339110 |
|  |  | LOC103339111 | RNA polymerase sigma factor sigB |
|  |  | LOC103339121 | uncharacterized LOC103339121 |
|  |  | LOC103339123 | aladin |
|  |  | LOC103339112 | alpha-L-arabinofuranosidase 1-like |
|  |  | LOC103339113 | alpha-L-arabinofuranosidase 1-like |
|  |  | LOC103339115 | histone-lysine N-methyltransferase, H3 lysine-9 specific SUVH1-like |
|  |  | LOC103339124 | crossover junction endonuclease MUS81-like |
|  |  | LOC103339135 | protein FAR1-RELATED SEQUENCE 5-like |
|  | 3340001~3570000 | LOC103339168 | vinorine synthase-like |
|  |  | LOC103339179 | wall-associated receptor kinase-like 3 |
|  |  | LOC103339169 | vinorine synthase-like |
|  |  | LOC103339171 | uncharacterized LOC103339171 |
|  |  | LOC103339172 | agamous-like MADS-box protein AGL82 |
|  |  | LOC107881678 | RNA-directed DNA polymerase homolog |
|  |  | LOC103339173 | ABC transporter A family member 1-like |
|  |  | LOC107881679 | uncharacterized LOC107881679 |
|  |  | LOC103339181 | serine carboxypeptidase-like 45 |
|  | 3670001~3840000 | LOC103339186 | uncharacterized LOC103339186 |
|  |  | LOC103339187 | microtubule-associated protein 70-2-like |
|  |  | LOC103339205 | probable LRR receptor-like serine/threonine-protein kinase At4g36180 |
|  |  | LOC103339206 | uncharacterized LOC103339206 |
|  |  | LOC103339207 | LRR receptor-like serine/threonine-protein kinase GSO1 |
|  |  | LOC103339204 | LRR receptor-like serine/threonine-protein kinase GSO2 |
|  |  | LOC103339188 | DNA gyrase subunit B, chloroplastic/mitochondrial |
|  |  | LOC103339191 | uncharacterized LOC103339191 |
|  |  | LOC103339190 | sulfoquinovosidase-like |
|  |  | LOC103339192 | pentatricopeptide repeat-containing protein At1g80270, mitochondrial-like |
|  |  | LOC103339193 | arginine/serine-rich coiled-coil protein 2 |
|  |  | LOC103339194 | F-box protein At2g26850-like |

Table S6. SNPs and candidate genes from GWAS analysis

| Chromosome | SNP location | Gene symbol | Gene description |
| --- | --- | --- | --- |
| LG1 (LG6) | 23819865 | LOC103344564 | GDSL esterase/lipase At1g09390-like |
| LG1 (LG6) | 24951867 | LOC103344713 | FRIGIDA-like protein 3 |
| LG1 (LG6) | 24951867 | LOC103344710 | 3-dehydroquinate dehydratase/shikimate Dehydrogenase, chloroplastic-like, transcript variant X1 |
| LG1 (LG6) | 23663072 | LOC103344276 | Importin subunit alpha-4 |
| LG1 (LG6) | 16082145 | LOC103333662 | PRA1 family protein D-like |
| LG2 (LG1) | 14235870 | LOC103321067 | 4-hydroxybenzoate geranyltransferase 2-like |
| LG2 (LG1) | 40321830 | LOC103324083 | Serine/threonine-protein kinase tricorner-like, transcript variant X1 |
| LG3 (LG4) | 1162655 | LOC103324577 | Cysteine-rich repeat secretory protein 38-like, transcript variant X1 |
| LG3 (LG4) | 1162655 | LOC107880642 | Putative receptor-like protein kinase At4g00960 |
| LG3 (LG4) | 1183704 | LOC103324685 | Putative receptor-like protein kinase At4g00960 |
| LG3 (LG4) | 7550849 | LOC103325630 | (E,E)-alpha-farnesene synthase-like |
| LG3 (LG4) | 23016869 | LOC103327201 | Uncharacterised |
| LG3 (LG4) | 923391 | LOC103324533 | E3 ubiquitin-protein ligase BRE1B-like |
| LG4 (LG3) | 10443038 | LOC103328489 | Uncharacterised |
| LG4 (LG3) | 11075246 | LOC103328542 | UPF0481 protein At3g47200-like |
| LG4 (LG3) | 11075246 | LOC103328524 | Uncharacterised |
| LG4 (LG3) | 11075246 | LOC103328527 | UPF081protein AT3G47200 |
| LG4 (LG3) | 15255454 | LOC103328924 | Persulphide dioxygenase ETHE1 homolog, mitochondrial-like |
| LG4 (LG3) | 15355284 | LOC103328939 | Transmembrane protein 56-like |
| LG4 (LG3) | 22817690 | LOC103330118 | Phenolic glucoside malonyltransferase 1-like |
| LG4 (LG3) | 24993869 | LOC103333537 | Mitochondrial import inner membrane translocase subunit TIM22-like |
| LG4 (LG3) | 24993869 | LOC103333536 | TRAF2 and NCK-interacting protein kinase-like |
| LG4 (LG3) | 14466649 | LOC103328851 | UDP-glucoronosyl and UDP-glucosyl transferase |
| LG5 (LG2) | 3304114 | LOC103330772 | Zinc finger protein 830（zf-met domain-containing protein） |
| LG5 (LG2) | 9114076 | LOC103331200 | Uncharacterised |
| LG5 (LG2) | 24713672 | LOC103333480 | WEB family protein At3g02930, chloroplastic-like |
| LG5 (LG2) | 24713672 | LOC103333481 | Uncharacterised |
| LG5 (LG2) | 24994015 | LOC103333536 | TRAF2 and NCK-interacting protein kinase-like |
| LG5 (LG2) | 2774830 | LOC103330732 | Putative disease resistance RPP13-like protein 1 |
| LG6 (LG8) | 6205819 | LOC103334971 | auxin-induced protein 15A |
| LG6 (LG8) | 9472624 | LOC103335480 | Uncharacterised protein AT2G9880 |
| LG6 (LG8) | 10993385 | LOC103335643 | Cyclase-associated protein 1 |
| LG6 (LG8) | 10993386 | LOC107881357 | Cyclic nucleotide-gated ion channel 1-like |
| LG6 (LG8) | 14348382 | LOC103335976 | Protein of unknown function (DUF563) |
| LG6 (LG8) | 19115110 | LOC103336351 | Mitochondrial substrate carrier family protein B-like |
| LG7 (LG5) | 2877993 | LOC103336859 | Heat shock factor protein HSF8 |
| LG7 (LG5) | 15777362 | LOC103338673 | Probable inactive receptor kinase At1g48480 |
| LG7 (LG5) | 2877993 | LOC103336959 | Uncharacterised protein At2g29880 |
| LG8 (LG7) | 12643836 | LOC103340471 | RNA polymerase I-specific transcription initiation factor RRN3 |
| LG8 (LG7) | 5026858 | LOC103339299 | Glucan endo-1,3-beta-glucosidase |
| LG8 (LG7) | 12729826 | LOC103340479 | AT-rich interactive domain-containing protein 6-like |
| LG8 (LG7) | 7864120 | LOC103339599 | Hypothetical protein PRUPE_ppa020531mg |
| LG8 (LG7) | 9252021 | LOC103339876 | Alpha carbonic anhydrase 4 |
| LG8 (LG7) | 12729826 | LOC103340480 | Protein FAR1-RELATED SEQUENCE 5-like |
| LG8 (LG7) | 9166740 | LOC103339798 | Expansin-A9-like |
| LG8 (LG7) | 7115480 | LOC103339526 | Uncharacterised |
| LG8 (LG7) | 7497201 | LOC103339564 | Fasciclin-like arabinogalactan protein 17 |
| LG8 (LG7) | 7865298 | LOC103339601 | Eukaryotic translation initiation factor 3 subunit C-like |
| LG8 (LG7) | 8973871 | LOC103339774 | Aspartic proteinase-like protein 2 |
| LG8 (LG7) | 9253586 | LOC103339810 | Alpha carbonic anhydrase 7-like |
| LG8 (LG7) | 9856887 | LOC103339940 | CDPK-related kinase 5 |
| Unplaced Scaffold | 62359 | LOC103341699 | Protein YLS9 |
| Unplaced Scaffold | 584873 | LOC103342194 | Carbon catabolite repressor protein 4 homolog 3 |
| Unplaced Scaffold | 29492 | LOC103343626 | Probable LRR receptor-like serine/threonine-protein kinase At3g47570 |

Note: LG numberings in bracket indicate the LG numberings in the Dirlewanger’s paper.
