# Supplementary figures and images for: Association between blooming time and climatic adaptation in Prunus mume
Source: Ecol Evol. 2019 Dec 20;10(1):292–306. doi: 10.1002/ece3.5894 (PMC6972806; doi:10.1002/ece3.5894)

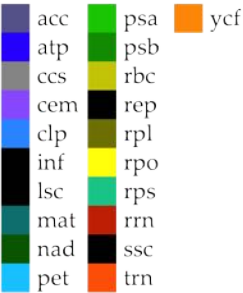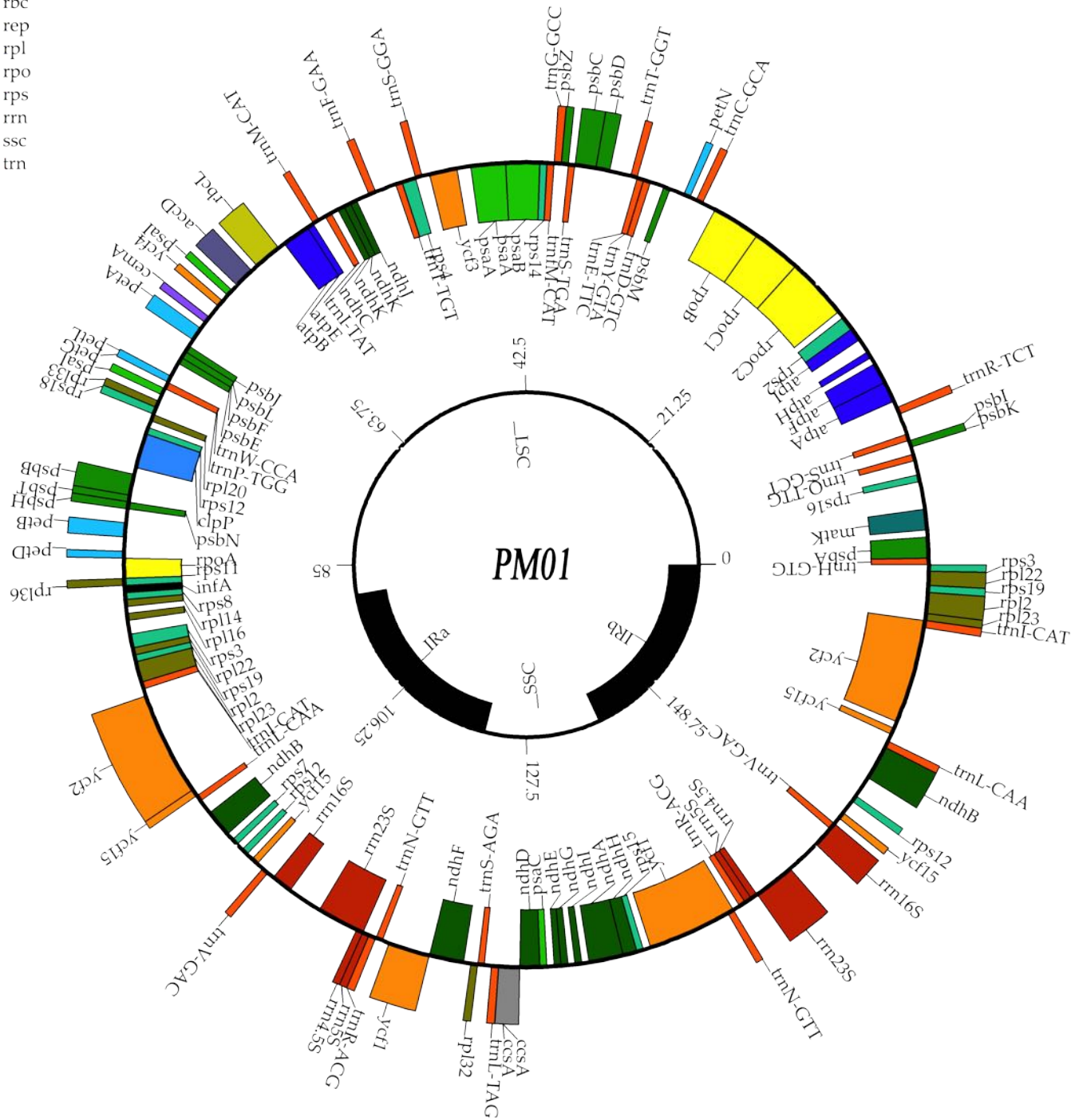

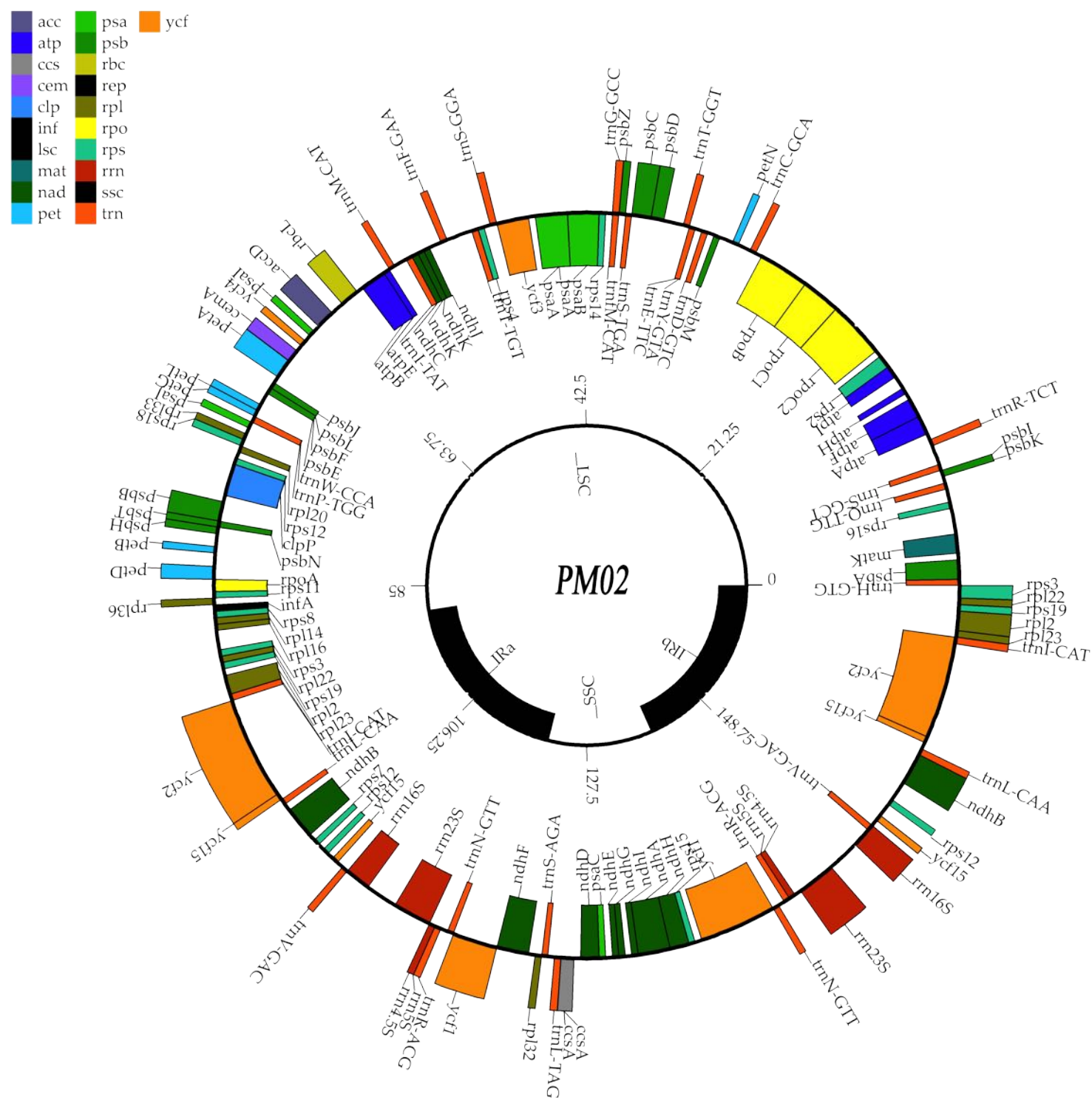

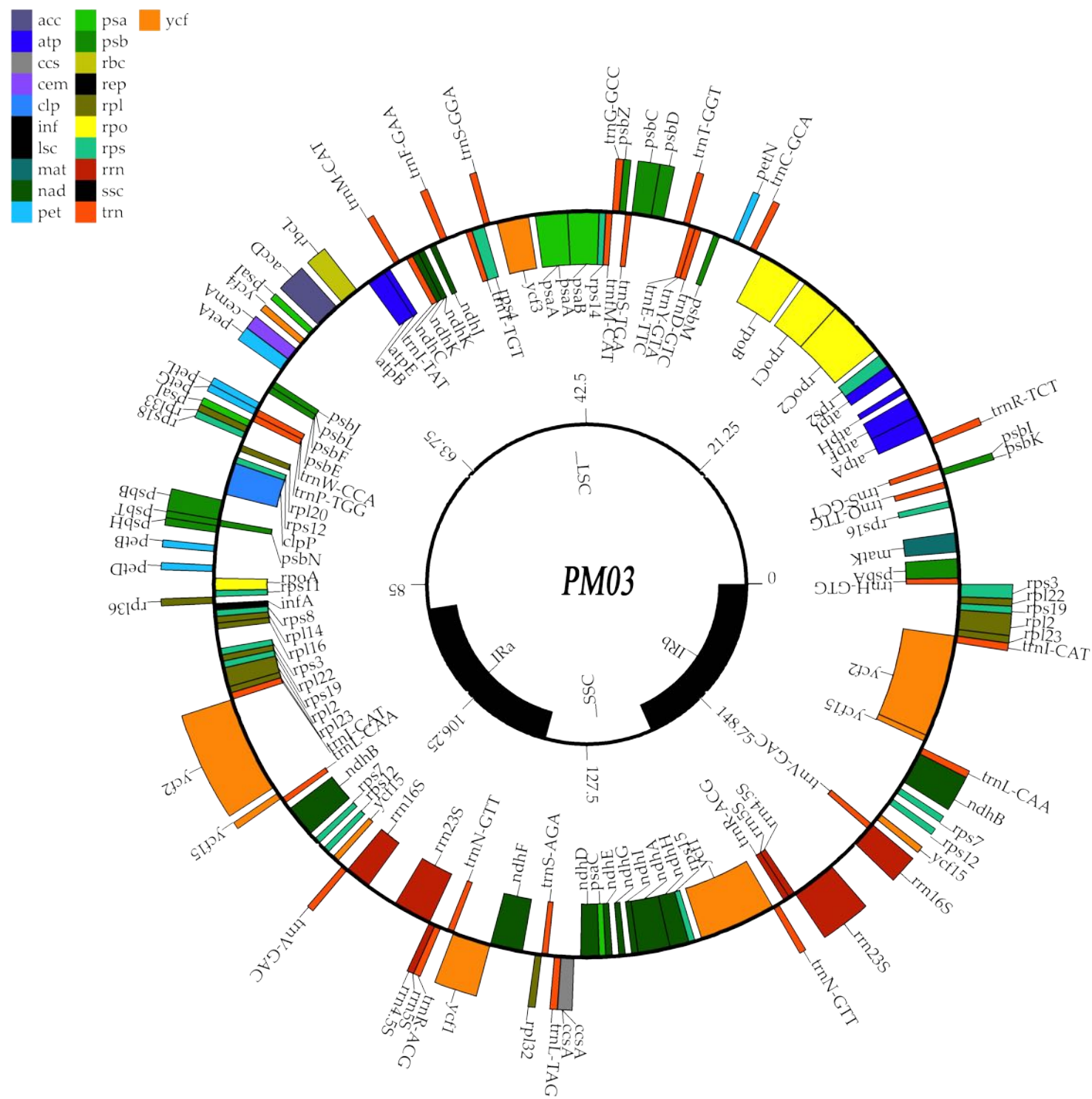

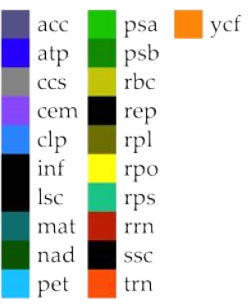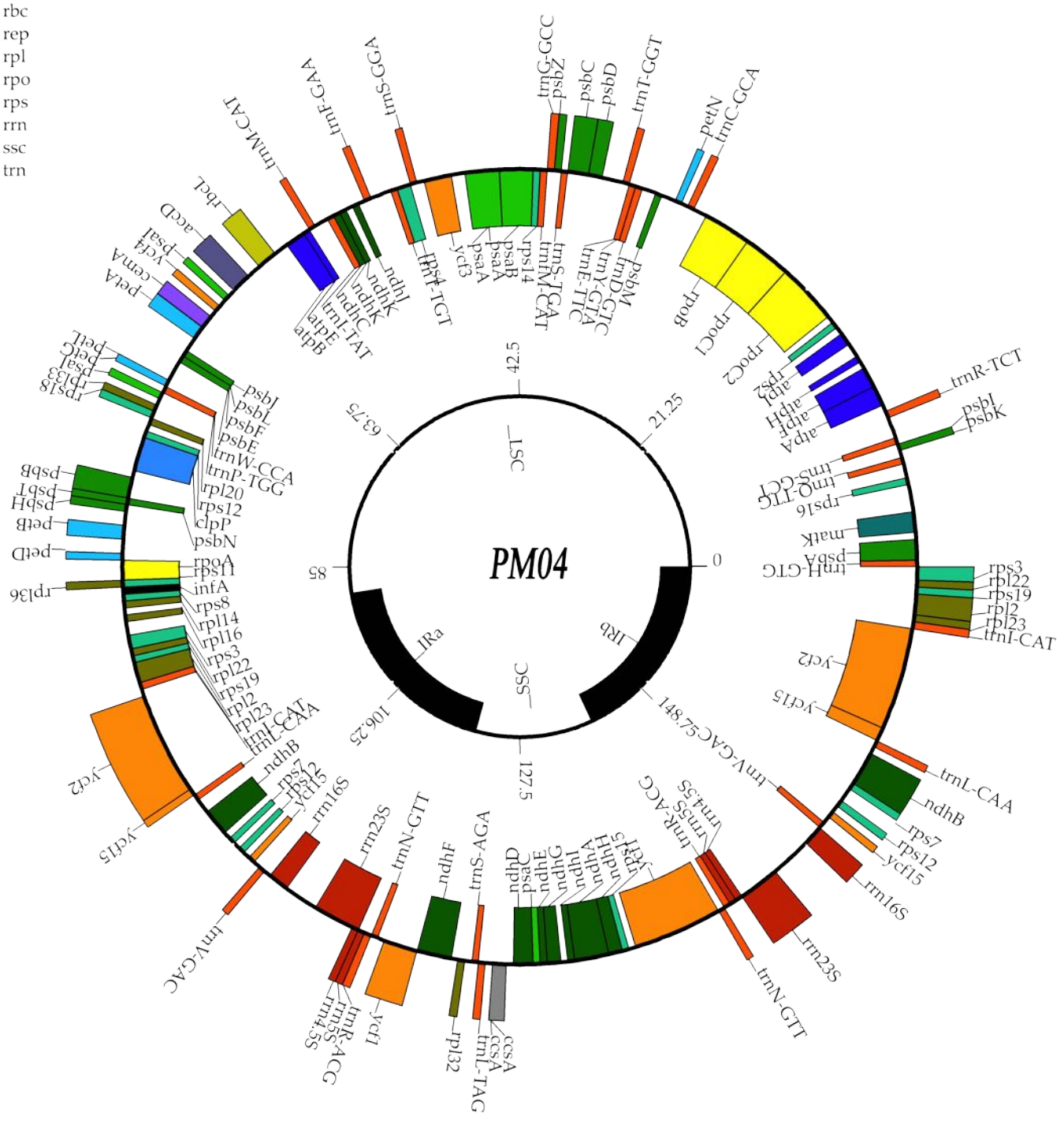

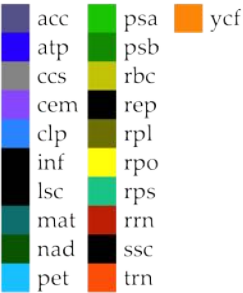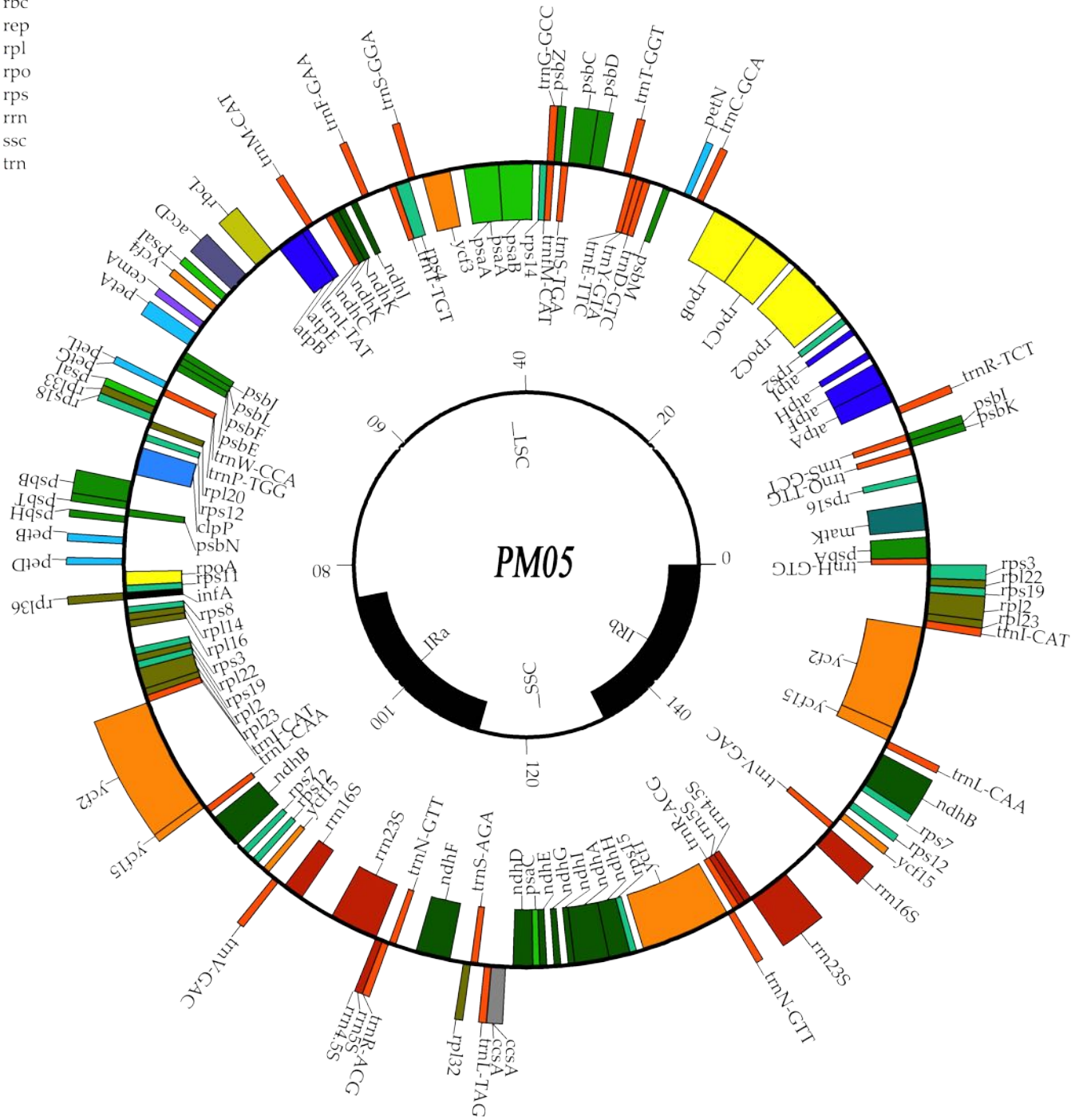

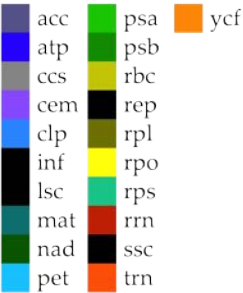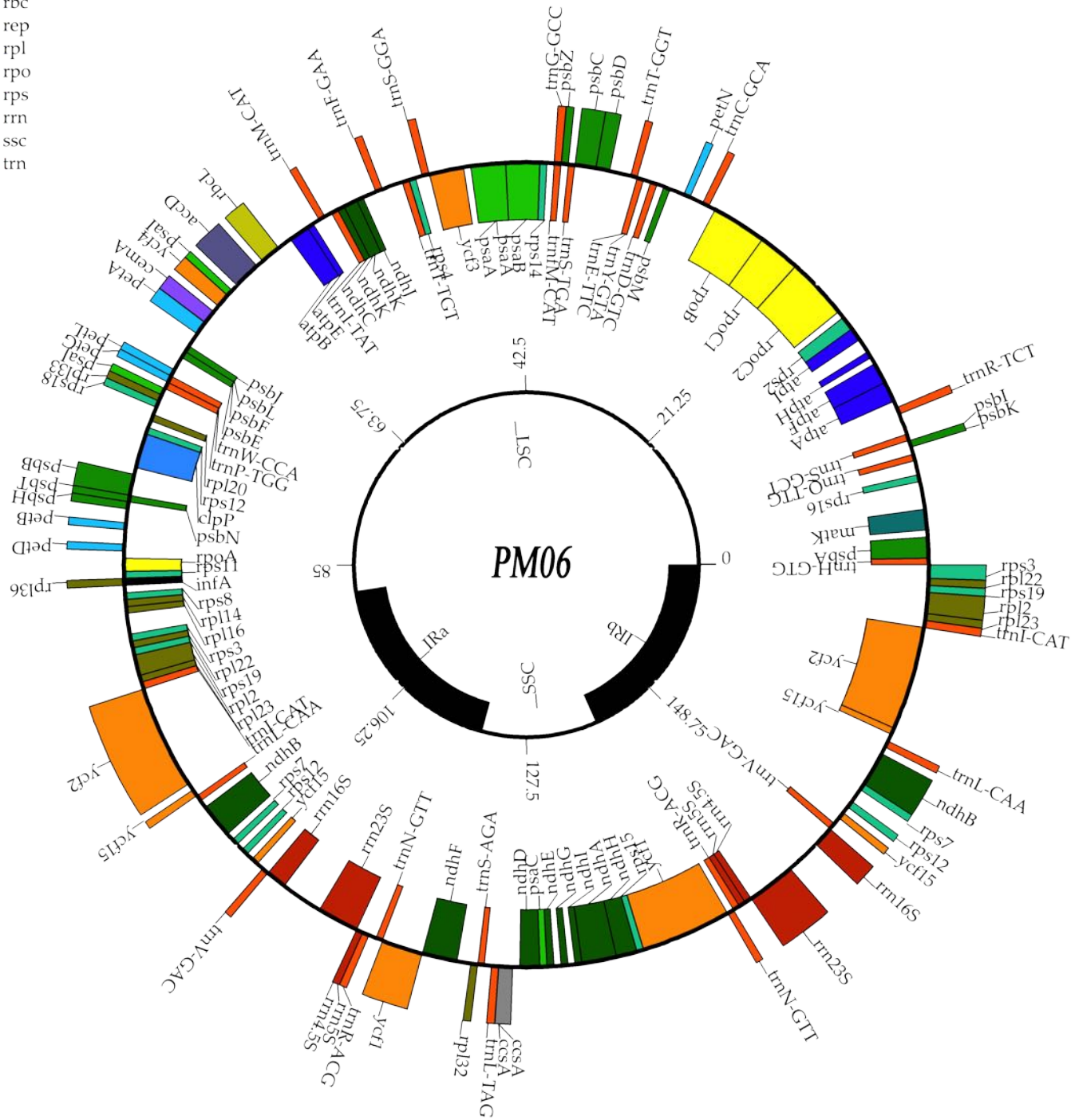

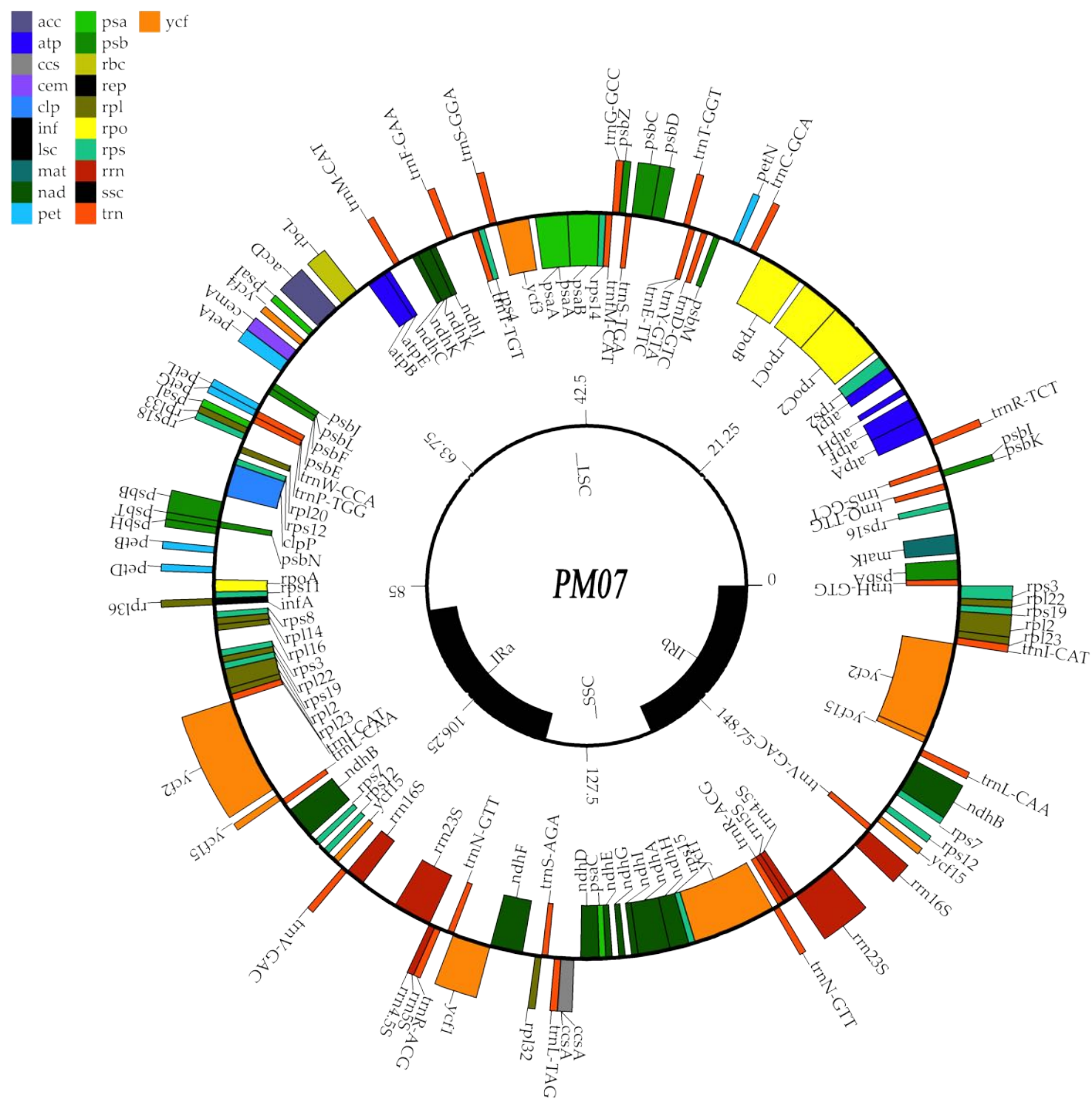

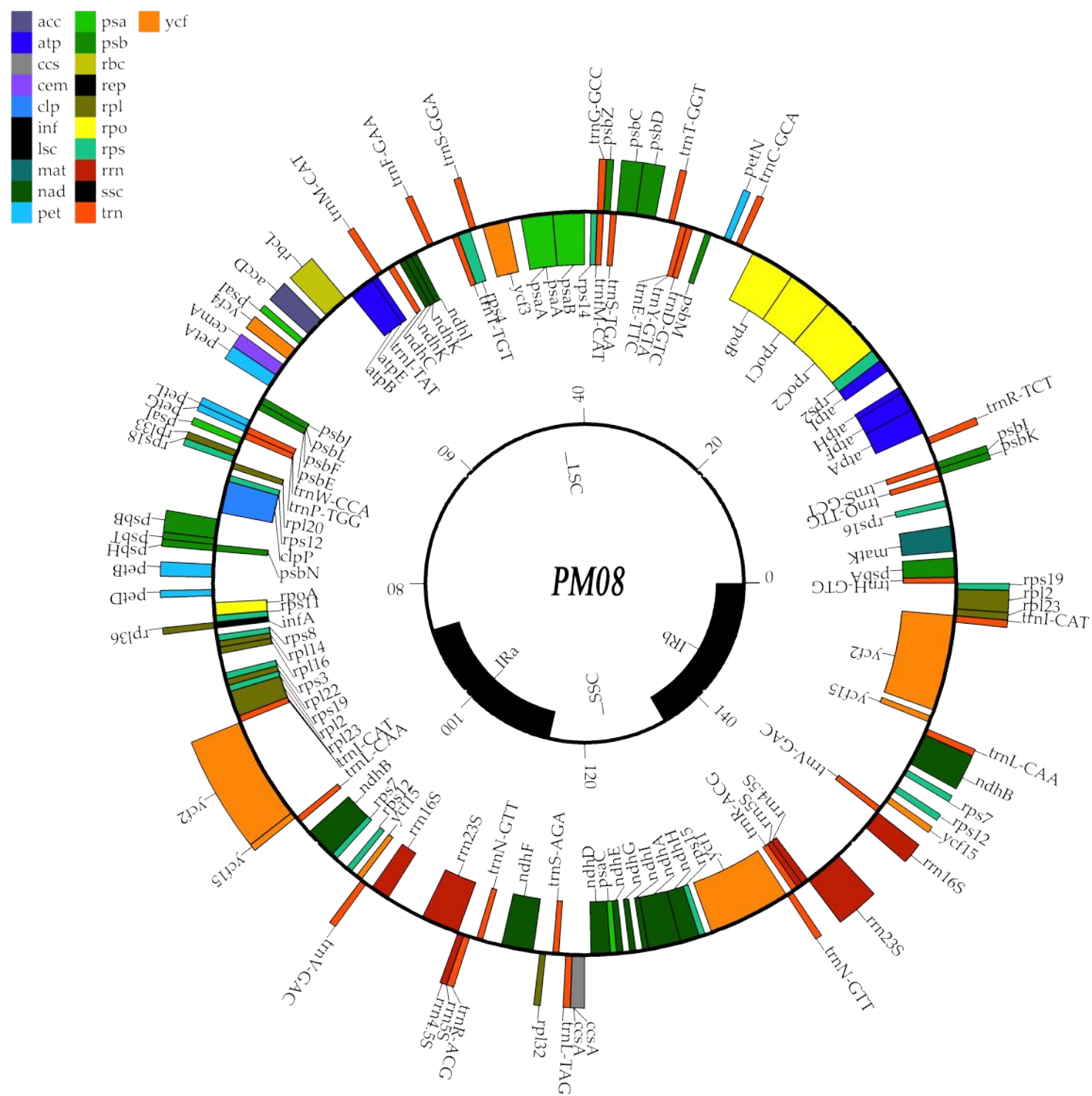

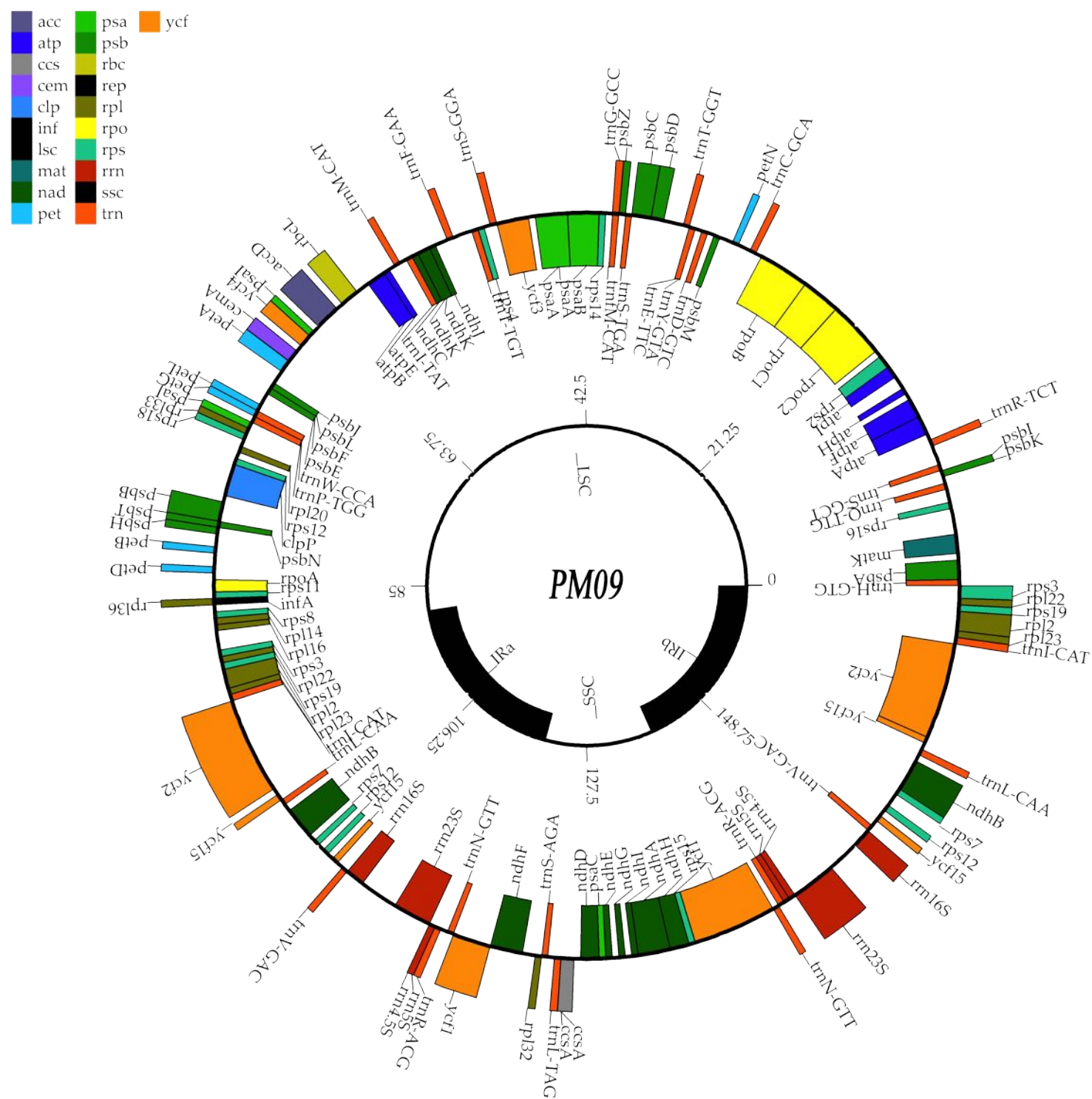

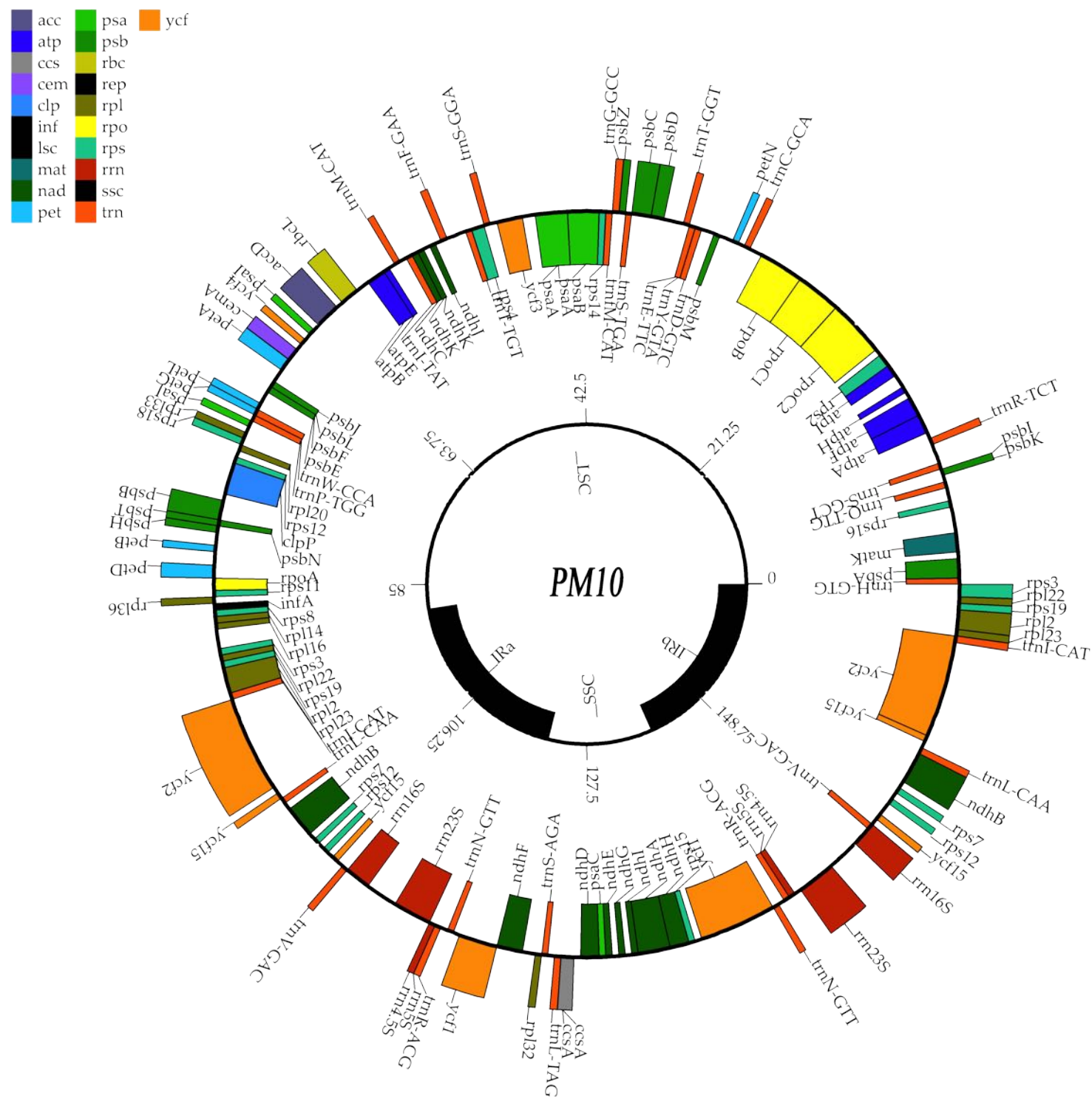

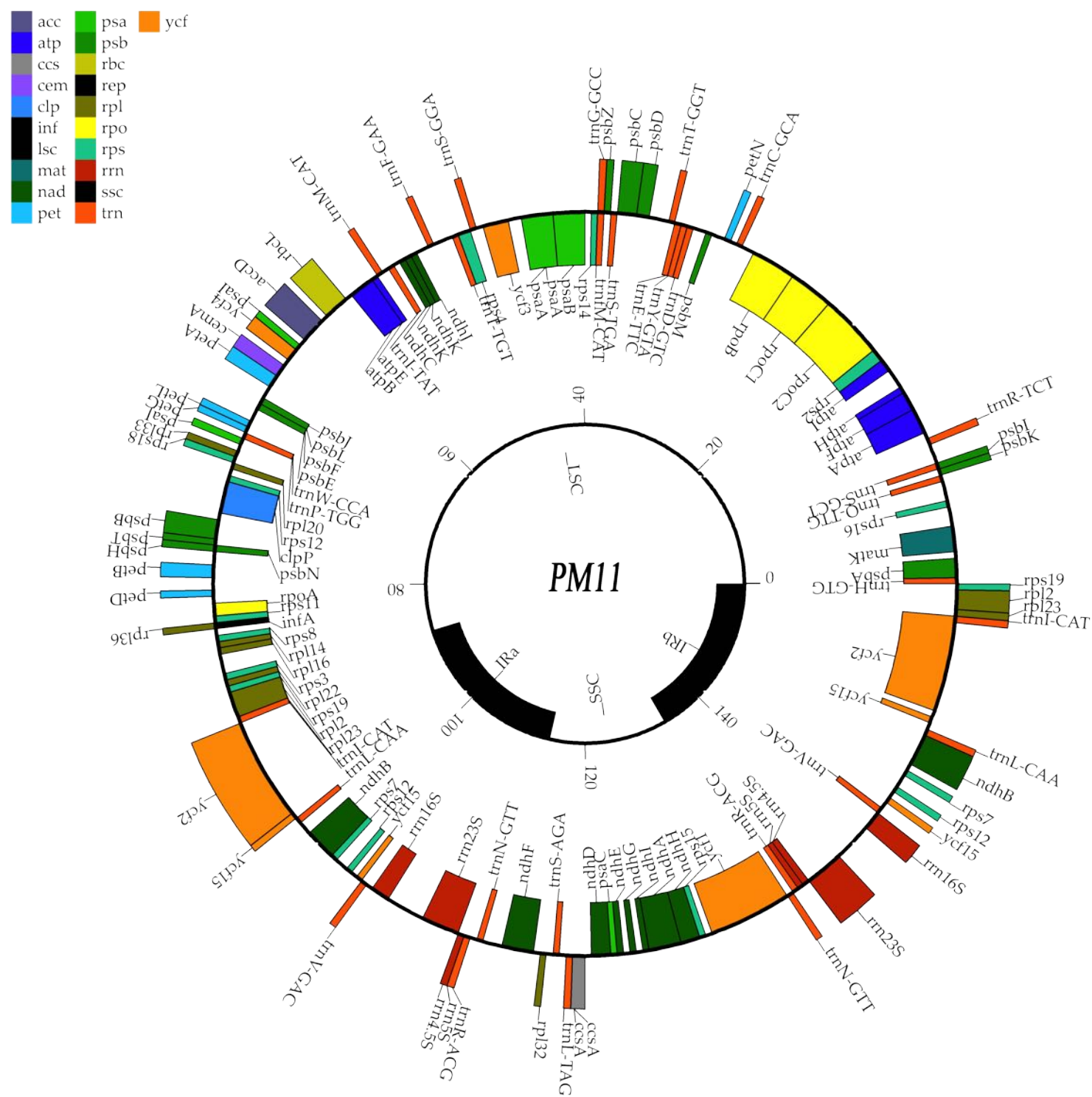

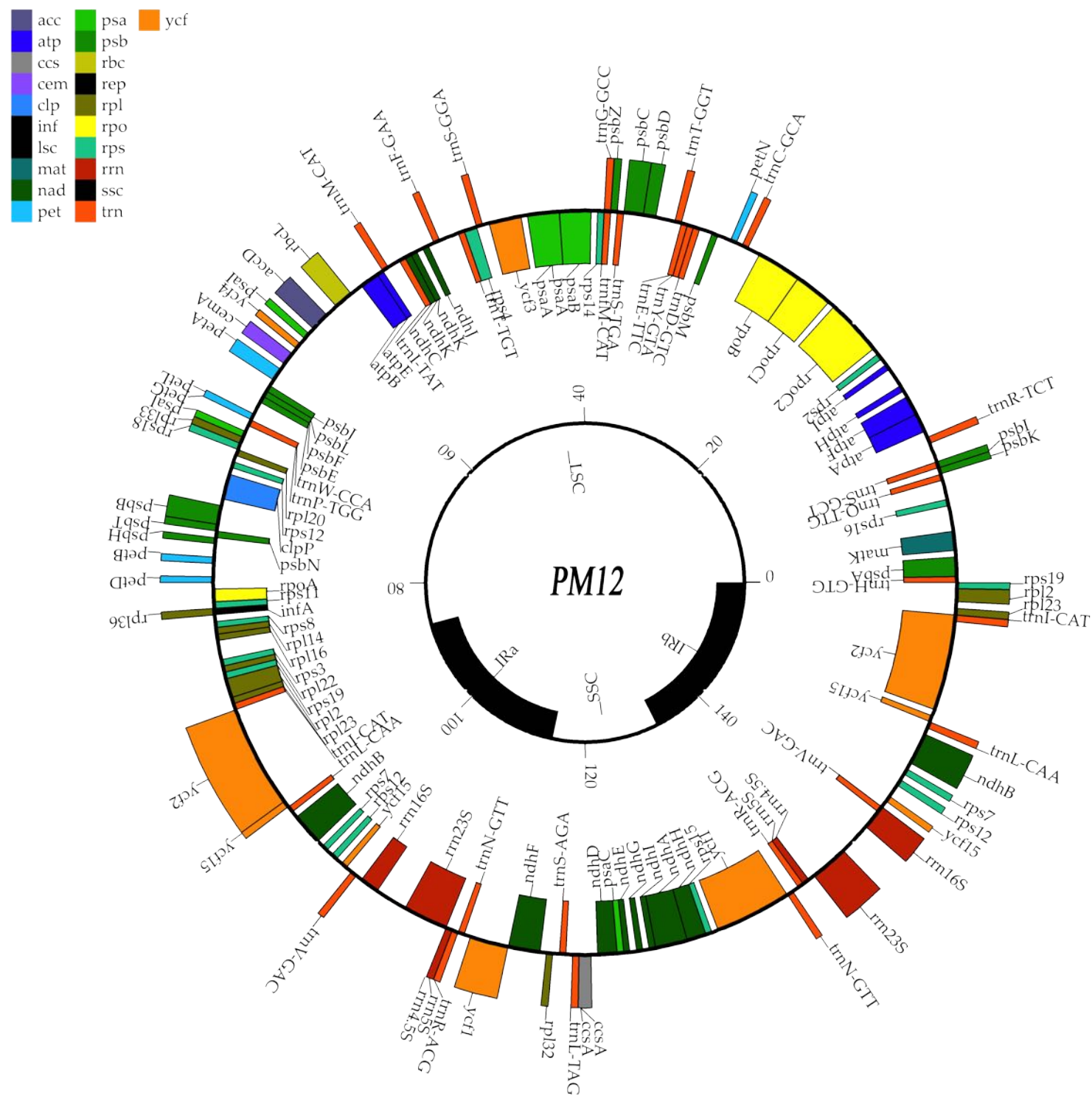

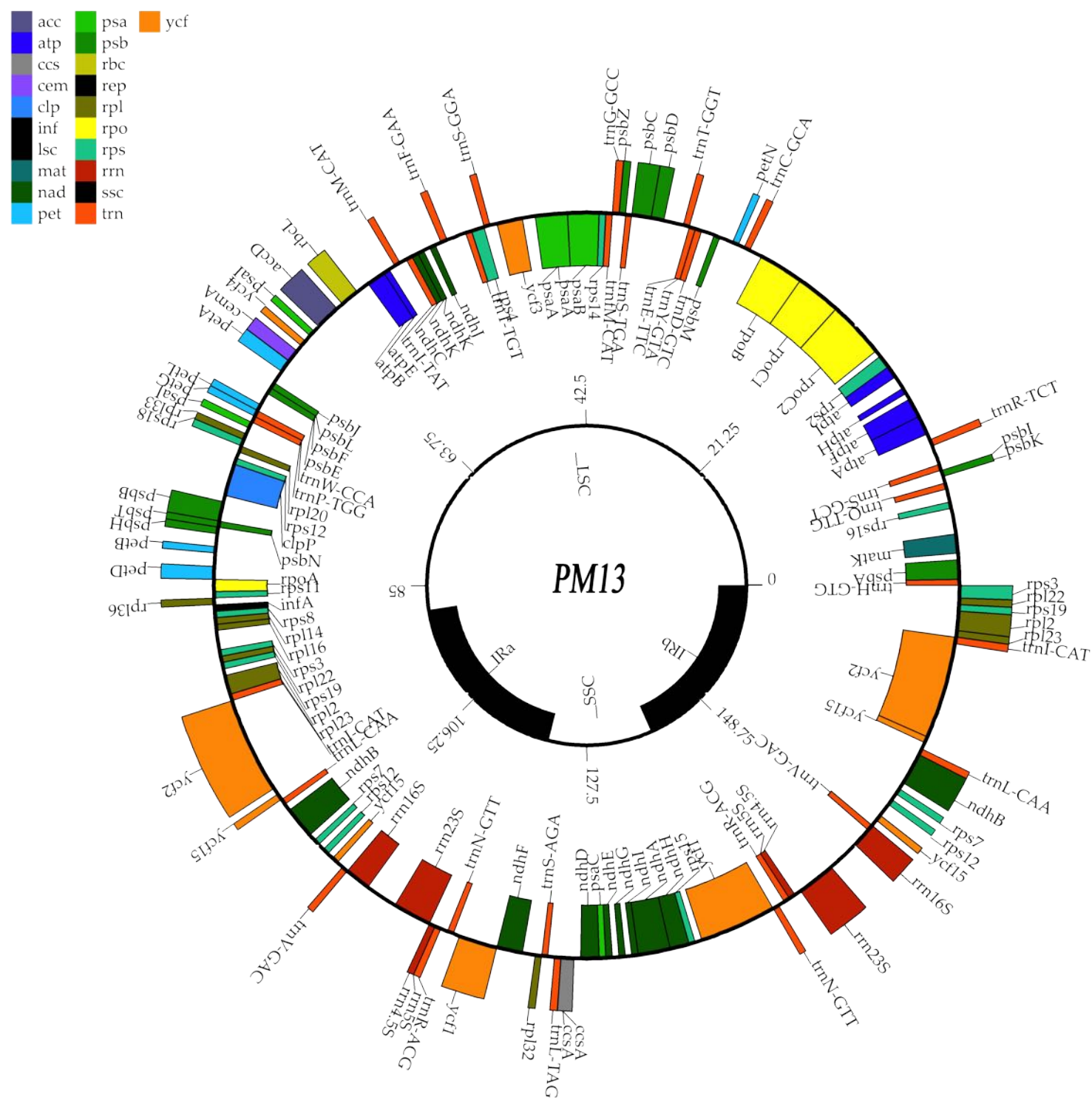

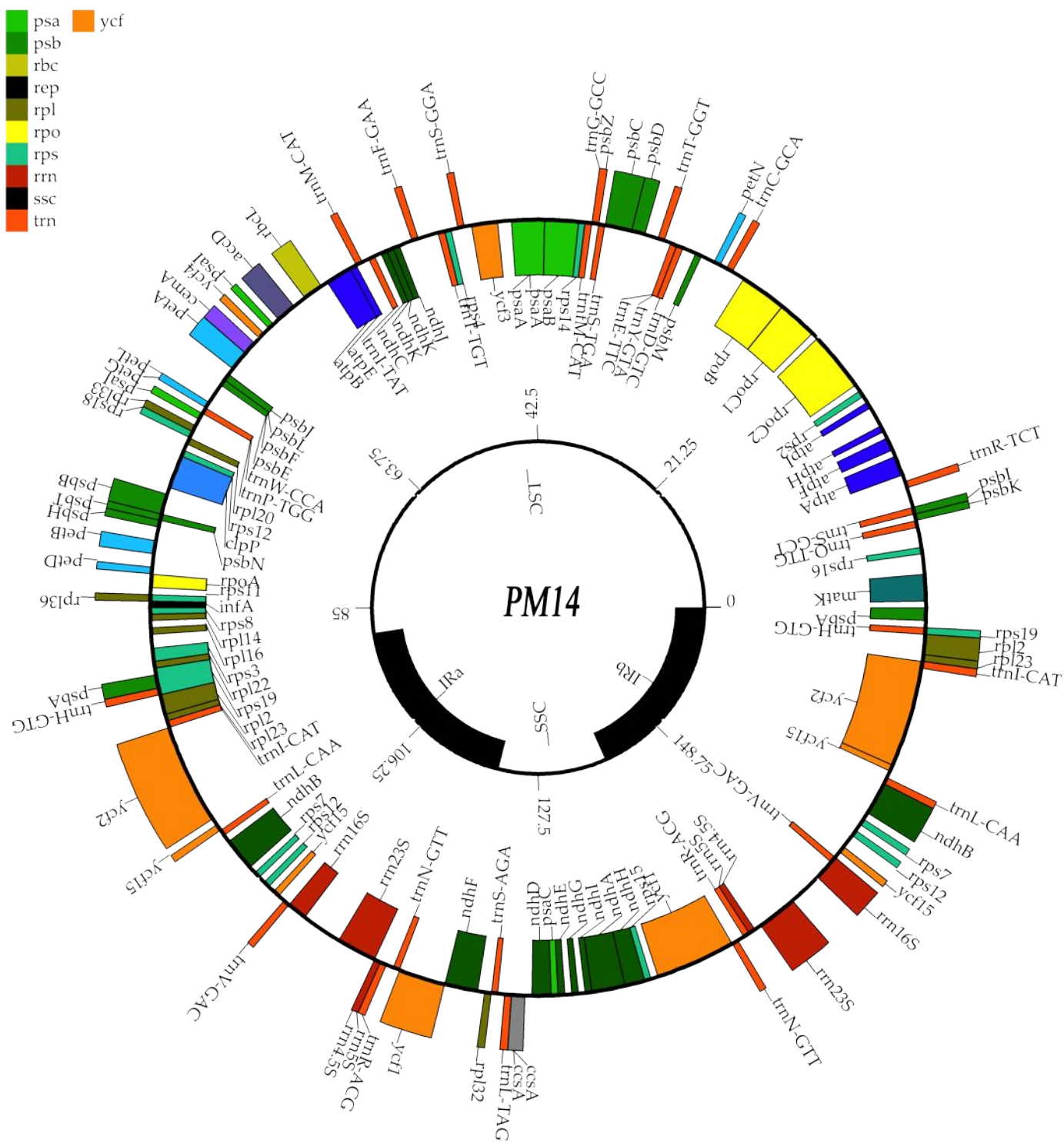

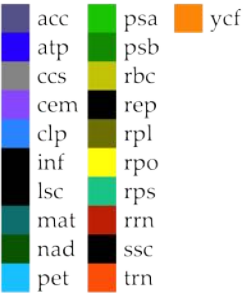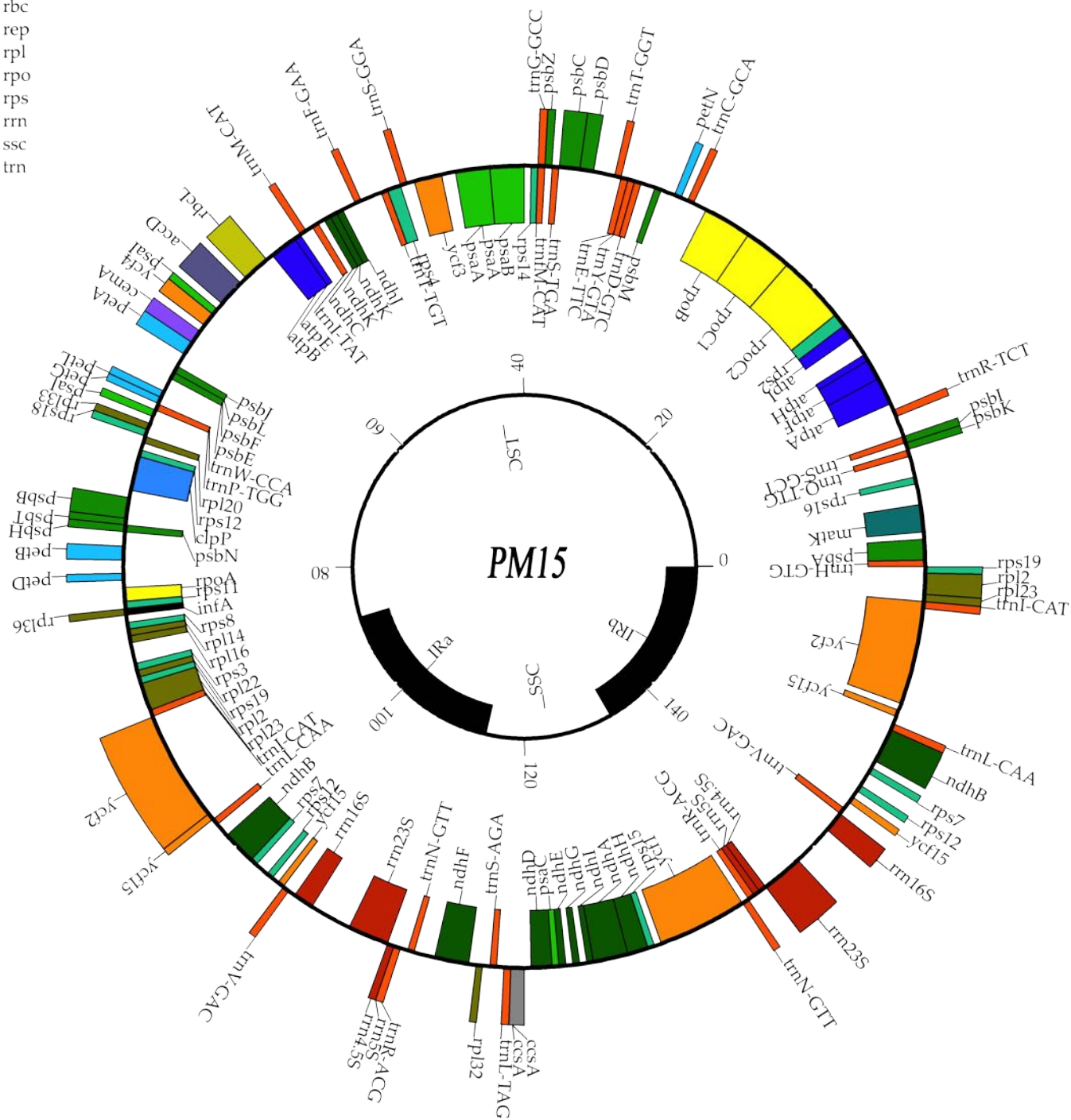

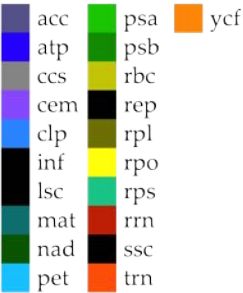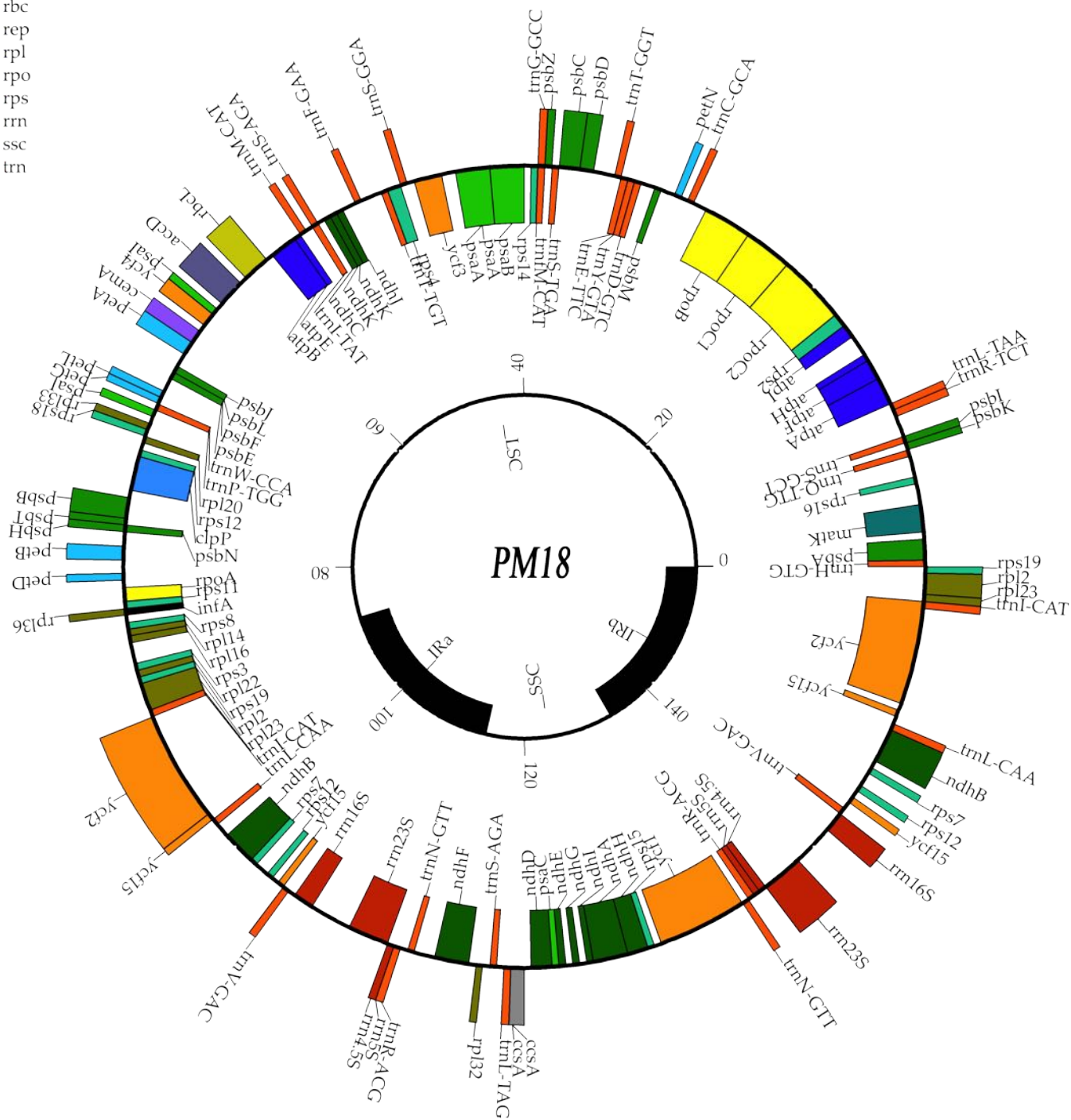

Supplement: Supplementary file 3 [file ECE3-10-292-s003.pdf]

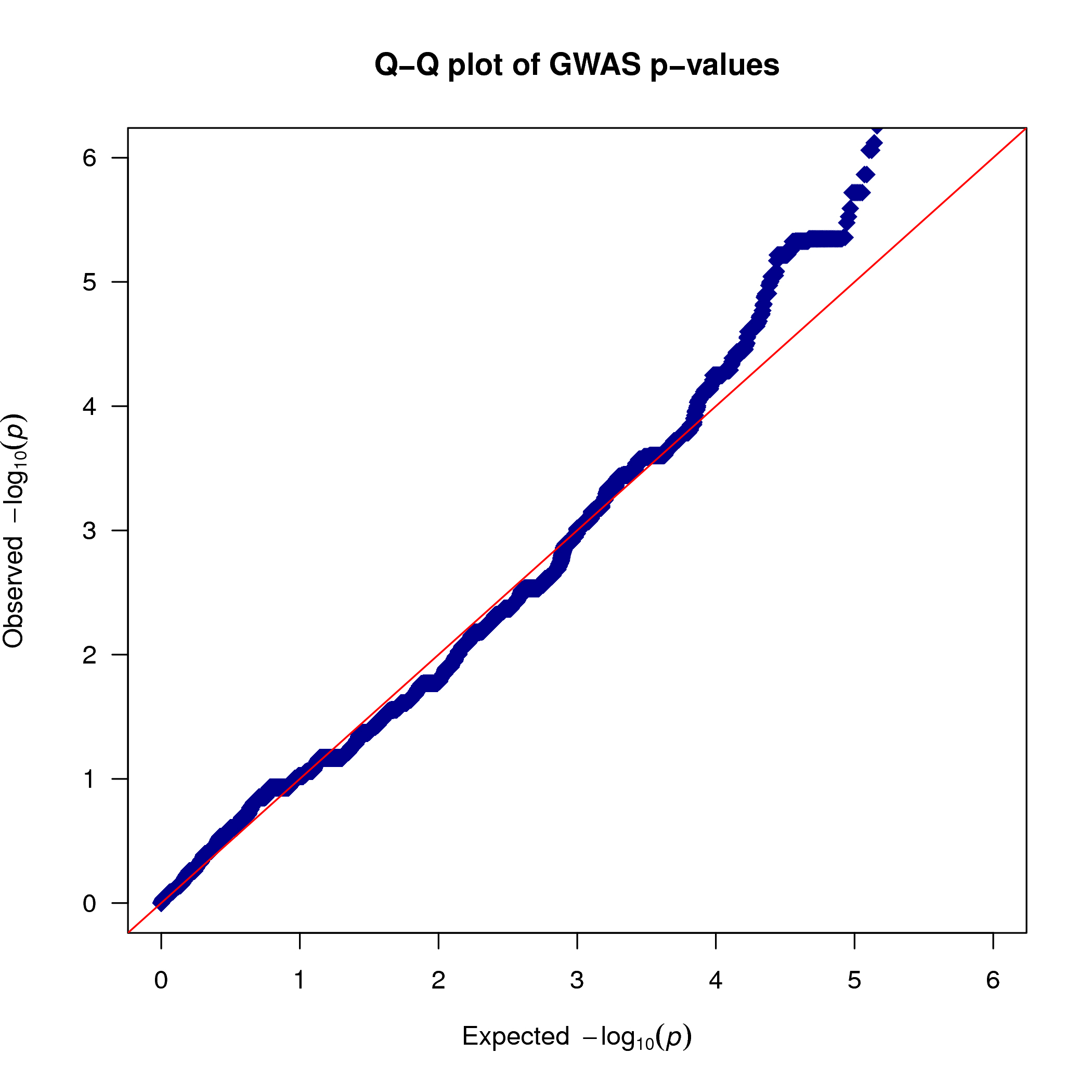

Supplement: Supplementary file 4 [file ECE3-10-292-s004.jpg]
